# Multi-Omics Integration Reveals Molecular Signatures Associated with Pork Quality Differences Between Liaodan Black and Xinjin Pigs

**DOI:** 10.3390/foods15203656

**Published:** 2026-10-15

**Authors:** Zhanpeng Jia, Yujinhe Tian, Yuqiao He, Xianjun Liu, Fubo Qiao, Wen Li, Jing Chen

**Affiliations:** College of Animal Science & Veterinary Medicine, Shenyang Agricultural University, Shenyang 110866, China

**Keywords:** Liaodan black pig, Xinjin pig, meat quality, multi-omics, metabolomics, pork

## Abstract

This exploratory study investigated meat quality differences and associated molecular characteristics between Liaodan Black (LDM) and Xinjin (XJM) pigs, two local breeds from Northeast China, using meat quality, fatty acid, and total amino acid measurements together with transcriptomic, proteomic, and untargeted metabolomic analyses of the longissimus thoracis et lumborum muscle (*n* = 3 per breed for all analyses). XJM pigs had higher intramuscular fat content and lower shear force, whereas LDM pigs had higher cooking yield. After correction for multiple testing, XJM pigs had higher oleic acid content. Total amino acid analysis showed higher serine, arginine, proline, methionine, and phenylalanine contents in XJM pigs, whereas isoleucine and leucine contents were higher in LDM pigs. Transcriptomic and proteomic analyses identified 5678 differentially expressed genes and 533 differentially abundant proteins, respectively, while untargeted metabolomic analysis revealed 622 differential metabolites based on putative annotations. These molecular differences were mainly associated with cytoskeletal organization, oxidative phosphorylation, protein turnover, amino acid metabolism, and central carbon metabolism. Transcriptome–proteome integration identified 258 shared differential features, of which 139 showed concordant changes at the mRNA and protein levels. Integrated analysis identified nine candidate molecules. After Benjamini–Hochberg correction, exploratory correlation analysis showed that GOT1 and GLO1 protein abundances were negatively associated with shear force and cooking yield, whereas ESD protein abundance was positively associated with IMF content. qPCR analysis of selected DEGs showed good agreement with RNA-seq results (r = 0.883, *p* < 0.001). Overall, the differences observed between the sampled LDM and XJM groups were mainly associated with lipid deposition, muscle structure-related processes, and energy metabolism.

## 1. Introduction

Pork quality is a complex phenotype involving tenderness, water-holding capacity, meat color, pH, intramuscular fat (IMF), and nutritional characteristics such as fatty acid and amino acid composition. Differences in genetic background and breeding objectives can influence muscle growth, fat deposition, and nutrient metabolism, resulting in breed-specific meat quality characteristics [1,2,3]. Liaodan Black (LDM) and Xinjin (XJM) are regionally developed pig breeds from Liaoning Province in Northeast China. LDM was developed from crosses between Liaoning Black dams and Duroc sires, followed by backcrossing and selection, and received national breed approval in 2021. XJM originated in the former Xinjin and Jin counties and is classified as a developed breed in the National Germplasm Bank of Domestic Animals. It is characterized by a robust body conformation, a predominantly black coat with white markings commonly occurring on the snout, tail tip, and lower limbs, and adaptability to regional production conditions.

Meat quality development involves coordinated changes at multiple molecular levels, including gene transcription, protein abundance, and small-molecule metabolism. Transcriptomics, proteomics, and metabolomics have therefore been increasingly applied to pork quality research to characterize molecular differences related to fat deposition, muscle growth, and meat quality [2,4]. However, most previous studies have focused on comparisons between indigenous and commercial lean-type pigs or on individual traits such as IMF and flavor. Studies integrating conventional meat quality traits, fatty acid and total amino acid composition, and transcriptomic, proteomic, and metabolomic data remain relatively limited.

Accordingly, this study compared IMF content, shear force, cooking yield, meat color, pH at 45 min postmortem, fatty acid composition, and total amino acid composition in the LTL muscle of LDM and XJM pigs managed under the same husbandry protocol. Transcriptomic, proteomic, and metabolomic data were integrated to identify molecular features associated with the observed meat quality patterns.

## 2. Materials and Methods

### 2.1. Sample Collection

LDM and XJM female pigs were raised at the Changtu Black Pig Breeding Farm in Liaoning Province, China. The two breeds were housed separately, with one pen per breed and 20 pigs in each pen, under the same husbandry protocol. During the finishing period, both breeds received the same farm-formulated diet, with ad libitum access to feed and drinking water. At 6 months of age, three pigs were randomly selected from the 20 animals reared within each breed for slaughter and sample collection. Complete pedigree records for the selected animals were not available; therefore, the degree of relatedness among the three pigs within each breed, including whether they were full siblings, half siblings, or unrelated, could not be determined. The biological sample size for each breed comparison was therefore three pigs per breed. The molecular analyses were interpreted as exploratory comparisons intended to identify candidate features for further investigation. Because pen housing was not replicated within breed, breed and pen effects could not be separated statistically; therefore, the comparisons were interpreted as exploratory differences between the sampled LDM and XJM groups rather than as independent estimates of breed effects. On an air-dry basis, the diet comprised 85.35% corn, 12.00% soybean meal, 0.60% dicalcium phosphate, 0.75% calcium phosphate, 0.30% sodium chloride, and 1.00% vitamin–mineral premix. The analyzed crude protein and calcium contents were 12.30% and 0.61%, respectively. The calculated digestible energy and available phosphorus levels were 13.81 MJ/kg and 0.27%, respectively. Individual body weight was recorded before slaughter, and the mean pre-slaughter body weights of LDM and XJM pigs were 118 and 120 kg, respectively. Pigs of both breeds were fasted for 12 h before loading for transport to a commercial abattoir. Upon arrival, they were held in lairage for 3 h in pens equipped with nipple drinkers. The pigs were then subjected to head-only electrical stunning (700 V, 1.25 A, 5 s), followed by exsanguination in a horizontal position within 30 s of stunning. LTL muscle samples were collected at the level of the last rib after slaughter. Samples designated for meat quality measurements were stored at 4 °C until analysis. Muscle pH was measured at approximately 45 min postmortem, whereas meat color, cooking yield, and Warner-Bratzler shear force were determined at 24 h postmortem. LTL muscle samples from the three selected pigs of each breed were used for meat quality measurements, fatty acid and total amino acid analyses, and transcriptomic, proteomic, and metabolomic analyses (*n* = 3 per breed). LTL muscle samples used for omics analyses were immediately snap-frozen in liquid nitrogen after collection, transported to the laboratory, and stored at −80 °C until further analysis.

### 2.2. Ethics Statement

The animal experimental protocol was approved by the Institutional Animal Care and Use Committee of Shenyang Agricultural University on 16 May 2025 (approval No. SNLL25051605). All experimental procedures were conducted in accordance with relevant animal welfare and ethical guidelines, with efforts made to minimize animal stress and unnecessary suffering.

### 2.3. Meat Quality Measurements

IMF content was determined by Soxhlet extraction as described by Xiong et al. [5]. Cooking yield and Warner-Bratzler shear force (WBSF) were determined according to the methods of Honikel [6] and Choi and Oh [7]. Briefly, LTL muscle samples were cut into approximately 2 cm-thick slices, placed in polyethylene bags, and heated in a water bath at 80 °C until the core temperature reached 71 °C. The samples were then cooled in ice water for 15 min, blotted dry to remove surface moisture, and weighed. Cooking yield was calculated as follows: CY (%) = cooked weight/raw weight × 100. The cooked samples were subsequently used for WBSF measurement. Six cylindrical cores (1.27 cm in diameter) were removed parallel to the muscle fiber direction and sheared perpendicular to the fibers using a Warner-Bratzler blade at a crosshead speed of 200 mm/min. The mean peak shear force of the six cores was recorded as the shear force of each sample. Meat samples were allowed to bloom in air for 30 min before color measurement. Meat color was measured using a CR-18 colorimeter (Beijing Color Reader Instrument Equipment Co., Ltd., Beijing, China) with an 8 mm aperture, D65 illuminant, and 0° viewing angle. CIE L* (lightness), a* (redness), and b* (yellowness) values were recorded at three different positions on each sample surface, and the mean value was used for statistical analysis. Muscle pH was measured approximately 45 min postmortem (pH_45_min) using a digital pH meter (pH-STAR, Matthäus GmbH & Co. KG, Eckelsheim, Germany).

### 2.4. Determination of Fatty Acid and Total Amino Acid Composition

Fatty acid composition of intramuscular lipids was determined by gas chromatography coupled with flame ionization detection (GC-FID) according to Zhang et al. [8], with slight modifications. Approximately 50 mg of freeze-dried muscle powder was extracted with chloroform–methanol (2:1, *v*/*v*), followed by base-catalyzed transesterification with KOH in methanol. Fatty acid methyl esters (FAMEs) were analyzed using an Agilent 7890B gas chromatograph (Agilent Technologies, Santa Clara, CA, USA) equipped with an Rt-2560 capillary column (100 m × 0.25 mm × 0.20 μm; Restek Corporation, Bellefonte, PA, USA). The oven temperature was maintained at 140 °C for 5 min, increased to 240 °C at 4 °C/min, and held for 15 min. Methyl nonadecanoate (C19:0; Sigma-Aldrich, St. Louis, MO, USA) was used as the internal standard and was added before sample extraction and derivatization. Individual FAMEs were identified by comparison of their retention times with those of a 37-component FAME standard mixture (Restek, Bellefonte, PA, USA). Quantification was performed using calibration curves prepared from the corresponding FAME standards, with analyte responses normalized to the C19:0 internal standard. Calibration curves were considered acceptable when R^2^ ≥ 0.995. Method performance was evaluated using spiked muscle samples, with an acceptable recovery range of 80–120% and a relative standard deviation (RSD) of ≤15% for replicate measurements. Fatty acid concentrations were converted to a fresh-weight basis according to the corresponding dry-to-fresh weight ratio and expressed as μg/g fresh muscle.

Total amino acid composition was determined by high-performance liquid chromatography (HPLC). Approximately 0.1–0.2 g of muscle was hydrolyzed with 5 mL of 6 mol/L HCl at 105 °C for 24 h, neutralized with 5 mL of 6 mol/L NaOH, and centrifuged at 4000 rpm for 10 min. An aliquot (0.5 mL) of the supernatant was mixed with 0.5 mL sodium bicarbonate buffer (pH 9.0) and 0.5 mL 2,4-dinitrofluorobenzene (DNFB), and derivatized at 60 ± 0.5 °C for 60 min in the dark. After addition of 3.5 mL phosphate buffer (pH 7.0), samples were filtered through a 0.22 μm membrane for HPLC analysis. An Agilent 1200 Series HPLC system (Agilent Technologies, Santa Clara, CA, USA) with a C18 column (250 mm × 4.6 mm, 5 μm) was used at 38 °C, with detection at 360 nm, a flow rate of 1.0 mL/min, and an injection volume of 20 μL. Mobile phases A and B were acetonitrile–methanol (90:10, *v*/*v*) and 0.02 mol/L phosphate buffer, respectively. The gradient was 14% A at 0 min, 40% at 39 min, 70% at 44 min, and 14% at 44.01–49 min. Amino acid contents were expressed as milligrams per gram of fresh muscle tissue (mg/g fresh weight).

### 2.5. Transcriptome Sequencing and Analysis

Total RNA was extracted from LTL muscle samples of LDM and XJM pigs using TRIzol^®^ reagent (Life Technologies, Carlsbad, CA, USA). RNA purity was assessed using a NanoDrop ND-2000 spectrophotometer (Thermo Scientific, Waltham, MA, USA), and RNA integrity was evaluated using an Agilent 2100 Bioanalyzer (Agilent Technologies, Santa Clara, CA, USA). Only RNA samples with an RNA integrity number (RIN) ≥ 7.0 were used for library construction. A total of 1.5 μg RNA per sample was used to construct sequencing libraries with the NEBNext^®^ Ultra™ RNA Library Prep Kit for Illumina^®^ (NEB, Ipswich, MA, USA) according to the manufacturer’s instructions. Library quality was assessed using an Agilent 2100 Bioanalyzer (Agilent Technologies, Santa Clara, CA, USA), and qualified libraries were sequenced on an Illumina NovaSeq 6000 platform (Illumina, Inc., San Diego, CA, USA) to generate 150 bp paired-end reads.

After removal of adaptor sequences and low-quality reads, sequencing quality was evaluated using the numbers of raw and clean reads, sequencing error rate, Q20, Q30, and GC content. For the six RNA-seq samples included in the present comparison, 42.67–53.92 million clean reads (6.29–7.96 Gb) were obtained per sample, with sequencing error rates of 0.02–0.03%, Q20 values of 98.10–98.59%, Q30 values of 94.13–95.34%, and GC contents of 44.13–45.79%. Clean reads were aligned to the pig (Sus scrofa) reference genome using HISAT2 (v2.1.0). The total mapping rates ranged from 95.99% to 96.93%, and uniquely mapped rates ranged from 92.13% to 93.47%. Detailed sample-level sequencing and alignment statistics are provided in Supplementary Table S2.

Gene-level read counts were generated using featureCounts (v1.5.0-p3), whereas FPKM values were calculated separately for descriptive representation of gene-expression levels. Differential-expression analysis was performed using DESeq2 (v1.42.0) based on raw gene-level counts and a negative-binomial model. The complete count matrix contained 36,085 genes, and no additional FPKM-based prefilter was applied before differential-expression testing. Genes lacking sufficient information for statistical testing were not classified as differentially expressed, and low-information genes were handled using the default independent-filtering procedure implemented in DESeq2. Sequencing-depth normalization and gene-wise dispersion estimation were performed within the DESeq2 framework, and dispersion estimates were evaluated using the plotDispEsts diagnostic function (Supplementary Figure S4). *p*-values were adjusted for multiple testing using the Benjamini–Hochberg correction procedure, and genes with an adjusted *p*-value < 0.05 were considered differentially expressed genes (DEGs).

GO and KEGG enrichment analyses of DEGs were performed using the clusterProfiler R package (v3.2.14). For KEGG enrichment analysis, Sus scrofa Ensembl gene identifiers were mapped to species-specific KEGG pathways (organism code: ssc). All genes with KEGG pathway annotations were used as the enrichment background. Pathway over-representation was evaluated using a hypergeometric test, followed by Benjamini–Hochberg correction for multiple testing. Pathways with an adjusted *p*-value < 0.05 were considered significantly enriched.

#### qPCR Analysis

The same LTL muscle samples used for RNA-seq analysis (*n* = 3 per breed) were used for qPCR analysis. Ten DEGs, including COL3A1, COL1A1, DES, TNNT3, MYH7, MYH4, NEB, TMOD4, MYL1, and THBS2, were selected for qPCR analysis, with three biological replicates for both LDM and XJM pigs. Total RNA was reverse-transcribed into cDNA using the PrimeScript™ RT Reagent Kit (Takara, Dalian, China). Gene-specific primers were designed using Primer Premier 5.0 software (PREMIER Biosoft International, Palo Alto, CA, USA), and the primer sequences are listed in Table 1. Real-time quantitative PCR was performed using SYBR Green PCR Master Mix (Vazyme, Nanjing, China) according to the manufacturer’s instructions. qPCR was performed on a SLAN-96 Real-Time PCR System (Hongshi Medical Technology, Shanghai, China). The reaction volume was 20 μL. The amplification program consisted of an initial denaturation at 95 °C for 5 min, followed by 40 cycles of denaturation at 95 °C for 15 s and annealing/extension at 60 °C for 1 min. Melting curve analysis was performed after amplification to assess amplification specificity. Each biological sample was analyzed in three technical replicates. GAPDH was used as the reference gene, and relative gene expression levels were calculated using the 2^−ΔΔCt^ method.

### 2.6. Protein Extraction and Proteomic Analysis

Proteins were extracted from LTL muscle samples using SDT buffer (4% SDS, 100 mM Tris-HCl, pH 7.6), and protein concentrations were determined using a BCA assay kit (Bio-Rad, Hercules, CA, USA). Equal amounts of protein were reduced with 10 mM dithiothreitol in 8 M urea at 37 °C for 60 min, alkylated with 50 mM iodoacetamide for 45 min in the dark, and digested overnight at 37 °C with trypsin (Promega Corporation, Madison, WI, USA; catalog no. V5113) at a protein-to-trypsin ratio of 50:1. The resulting peptides were desalted using C18 tips, eluted with 90% acetonitrile, lyophilized, and reconstituted in 20 μL of 0.1% formic acid. After centrifugation at 14,000× *g* for 20 min at 4 °C, 300 ng of peptides was subjected to LC-MS/MS analysis using a U3000 system coupled to an Orbitrap Astral mass spectrometer with a NanoSpray Flex ion source (Thermo Fisher Scientific, Waltham, MA, USA). Mobile phases A and B consisted of 0.1% formic acid in water and 80% acetonitrile containing 0.1% formic acid, respectively. Peptides were separated at 2500 nL/min using the following gradient of mobile phase B: 4% at 0 min, 8% at 0.1 min, 28% at 5.1 min, and 99% at 7.2–8.0 min. Data were acquired in DIA positive-ion mode over an *m*/*z* range of 380–980, with an MS1 resolution of 240,000 at *m*/*z* 200, an MS2 accumulation time of 3 ms, a spray voltage of 1.9 kV, and a cycle time of 0.6 s. Protein identification and quantification were performed using DIA-NN (v1.9.1) with a species-specific Sus scrofa protein sequence database [9]. Trypsin/P was specified as the proteolytic cleavage rule, allowing up to two missed cleavages and peptide lengths of 7–30 amino acids. Carbamidomethylation of cysteine was specified as a fixed modification, whereas methionine oxidation and protein N-terminal acetylation were specified as variable modifications. Identification confidence was controlled at a false discovery rate (FDR) of 1% at both the peptide-precursor and protein levels. No external spectral library was used in this analysis. Protein quantities were retained only for proteins passing the DIA-NN filtering criteria using the “Q-value sparse” mode. Protein abundances were median-normalized, and missing values were imputed using the K-nearest neighbor algorithm. DAPs were identified using Student’s *t*-test with *p* < 0.05 and fold change > 1.5. GO and KEGG enrichment analyses were subsequently performed using hypergeometric tests, with all identified proteins used as the background set. *p*-values from the enrichment analyses were adjusted for multiple testing using the Benjamini–Hochberg correction method. The mass spectrometry proteomics data have been deposited to the ProteomeXchange Consortium via the PRIDE partner repository with the dataset identifier PXD084581. The dataset is currently private during peer review, and reviewer access credentials have been provided separately through the journal submission system.

### 2.7. Metabolite Extraction and Metabolomic Analysis

Metabolites were extracted from tissue samples according to Warren et al. [10]. Metabolomic analysis was performed using a Vanquish UHPLC system coupled to an Orbitrap Exploris 120 mass spectrometer equipped with an electrospray ionization source (Thermo Fisher Scientific, Waltham, MA, USA). Chromatographic separation was performed using an ACQUITY UPLC HSS T3 column (2.1 × 100 mm, 1.8 μm; Waters, Milford, MA, USA) at 40 °C, with a flow rate of 0.3 mL/min and an injection volume of 5 μL. In positive-ion mode, mobile phases A and B were 0.1% formic acid in water and 0.1% formic acid in acetonitrile, respectively; formic acid was purchased from TCI (Shanghai, China). In negative-ion mode, mobile phases A and B were 5 mM ammonium formate in water and acetonitrile, respectively; ammonium formate was purchased from Sigma-Aldrich (Shanghai, China). The gradient was 10% B at 0–1 min, 10–98% B at 1–5 min, 98% B at 5–6.5 min, 98–10% B at 6.5–6.6 min, and 10% B at 6.6–8 min. Mass spectrometric data were acquired in both positive- and negative-ion modes with spray voltages of 3.50 and −2.50 kV, respectively. The sheath gas and auxiliary gas were set to 40 and 10 arbitrary units, respectively, and the capillary temperature was 325 °C. Full MS scans were acquired over *m*/*z* 100–1000 at a resolution of 60,000. MS/MS spectra were acquired in ddMS2 mode at a resolution of 15,000 with a normalized HCD collision energy of 30%. The top four precursor ions were selected for fragmentation, with dynamic exclusion enabled.

Raw data were converted to mzXML format using MSConvert (v3.0.8789) in the ProteoWizard package. Peak extraction, retention-time correction, and alignment were performed using XCMS (v3.12.0) in R (version 4.5.3; R Foundation for Statistical Computing, Vienna, Austria) with bw = 2, ppm = 15, peakwidth = c(5, 30), mzwid = 0.015, mzdiff = 0.01, and method = centWave. An extraction blank was prepared using the same extraction procedure as the biological samples without the addition of tissue and was analyzed by LC–MS in both positive- and negative-ion modes. The relative acquisition order of the biological samples and extraction blanks listed in Supplementary Table S4 was determined from the acquisition timestamps recorded in the corresponding raw LC–MS files. Peak areas were normalized using total peak-area normalization separately for positive- and negative-ion modes before subsequent statistical analysis. No additional inter-batch correction was applied. No independent pooled-QC injection series was included, and no QC-based signal-drift correction or feature filtering based on QC-RSD thresholds was performed.

Metabolites were putatively annotated based on accurate mass, molecular formula and adduct information, and MS/MS spectral matching against HMDB, MassBank, KEGG, LIPID MAPS, mzCloud, and the Panomix in-house database, with a mass tolerance of <30 ppm. Because authentic chemical standards were not analyzed in the present study, no Level 1 metabolite identifications are claimed. Annotations supported by MS/MS spectral matching to reference databases were regarded as putative annotations consistent with MSI Level 2 confidence. However, a uniform MSI confidence level was not retrospectively assigned to all 1844 metabolite annotations because compound-specific confidence levels were not available in the original analytical report. Prior to multivariate statistical analysis, the processed data were autoscaled. PCA, PLS-DA, and OPLS-DA were performed using the ropls package (v1.22.0) in R. PCA was used for unsupervised visualization of sample clustering, whereas OPLS-DA was applied as a supervised multivariate approach for identifying metabolites contributing to group discrimination. The supervised models were evaluated using 100 permutation tests. Putatively annotated metabolites satisfying both a nominal *p*-value < 0.05 in the two-sided Student’s *t*-test and an OPLS-DA-derived VIP value > 1 were considered differential metabolites. The nominal *p*-values used for differential-metabolite screening were not adjusted for multiple testing. The OPLS-DA score plots and corresponding permutation-test results are provided in Supplementary Figure S3. Hierarchical clustering of the differential metabolites was performed using the pheatmap package (v1.0.12) in R after scaling the metabolite-abundance matrix, with bidirectional clustering of samples and metabolites. Pathway enrichment analysis was performed using a hypergeometric test, with *p*-values adjusted for multiple testing using the Benjamini–Hochberg correction method. Pathway topology analysis was based on betweenness centrality, and KEGG Mapper was used for pathway mapping and visualization. The raw LC–MS metabolomics data acquired in positive- and negative-ion modes, together with the associated sample and assay metadata and metabolite annotation tables, have been deposited in the MetaboLights repository under study accession MTBLS15873.

### 2.8. Multi-Omics Integration and Association Analyses

DEGs and DAPs were matched by gene symbol after excluding DEGs without an available gene symbol. Genes showing changes in the same direction at the transcript and protein levels were retained for further analysis. Among these genes, candidates encoding metabolic enzymes were screened by individually examining the corresponding reaction records in the Rhea database [11]. The substrates and products annotated in these records were matched against the differential metabolites selected in this study, and the resulting enzyme–metabolite links were used to select candidate genes, their corresponding proteins, and associated metabolites for subsequent integrated analysis. Spearman correlation analyses were then performed separately between candidate-gene transcript abundances and the relative abundances of the selected metabolites, and between the abundances of the corresponding proteins and the relative abundances of the same metabolites. The resulting correlation matrices were visualized as heatmaps. Mantel tests were performed in R to assess associations between the selected transcript, protein, and metabolite profiles and individual meat quality traits. The results were visualized using the linkET package alongside a Spearman correlation matrix of the meat quality traits. Line widths and colors represented Mantel’s r and *p*-value categories, respectively. Spearman correlation analysis was then performed to construct a correlation network among candidate molecules, differential metabolites, and meat quality traits. *p*-values from all pairwise correlations evaluated for network construction were adjusted for multiple testing using the Benjamini–Hochberg correction procedure. Only correlations with |Spearman’s ρ| ≥ 0.8 and an adjusted *p*-value < 0.05 were retained in the final network.

### 2.9. Statistical Analysis

Statistical analyses of meat quality traits, fatty acid composition, total amino acid composition, and qPCR data were performed using GraphPad Prism 9.0.0 (GraphPad Software, La Jolla, CA, USA). Meat quality traits, fatty acid contents, and total amino acid contents are presented as means ± standard deviations (SD). For qPCR, error bars for log_2_(fold change) values represent propagated standard errors calculated from three biological replicates per breed. Between-group differences in meat quality traits, fatty acid composition, and total amino acid composition were evaluated using two-sided independent-samples Student’s *t*-tests. For fatty acid and amino acid analyses, *p*-values were adjusted using the Benjamini–Hochberg procedure, separately across the 18 fatty acid comparisons and the 17 amino acid comparisons. Hedges’ g was calculated from the individual observations as a standardized mean difference with a small-sample correction, and 95% confidence intervals (CIs) for Hedges’ g were obtained using the noncentral t distribution. Hedges’ g was calculated as LDM minus XJM; therefore, positive values indicate higher values in LDM, whereas negative values indicate higher values in XJM. For fatty acid and amino acid analyses, statistical significance was defined as an adjusted *p*-value < 0.05. For meat quality traits, statistical significance was defined as a two-sided *p*-value < 0.05. Relative gene expression levels in the qPCR analysis were calculated using the 2^−ΔΔCt^ method, and Pearson correlation analysis was performed between the log_2_(fold change) values obtained by qPCR and RNA-seq for the 10 selected DEGs. The individual measurements underlying the meat-quality, fatty-acid, and amino-acid analyses are provided in Supplementary Table S1. To facilitate verification of the amino acid results presented in Table 3, the individual measurements for all 17 amino acids from three LDM and three XJM pigs, together with their corresponding laboratory sample identifiers, are additionally provided in Supplementary Table S3A. Supplementary Table S3B documents the recalculation of group means, standard deviations, nominal *p*-values, and Benjamini–Hochberg-adjusted *p*-values based on these same measurements.

## 3. Results

### 3.1. Meat Quality Characteristics of the LTL Muscle in LDM and XJM Pigs

**Figure 1:**
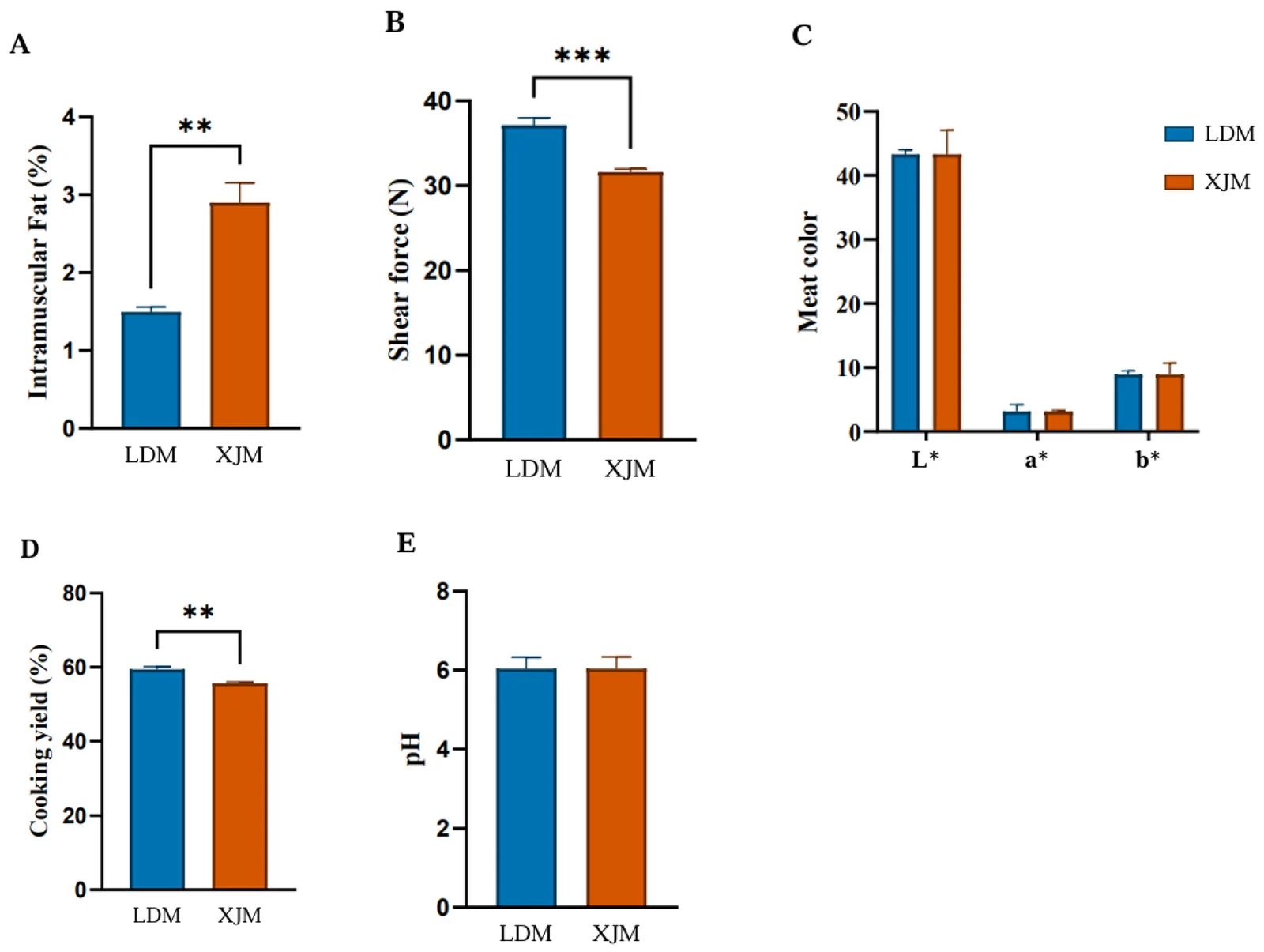
Comparison of meat quality traits in the LTL muscle between Liaodan Black (LDM) and Xinjin (XJM) pigs. (**A**) Intramuscular fat (IMF) content; (**B**) Warner-Bratzler shear force (WBSF); (**C**) meat color; (**D**) cooking yield; (**E**) pH_45_min. Data are presented as the mean ± SD (*n* = 3 pigs per breed). ** *p* < 0.01; *** *p* < 0.001.

To compare meat quality traits between LDM and XJM pigs, IMF content, shear force, meat color, cooking yield, and pH at 45 min postmortem were measured in the LTL muscle (Figure 1). The XJM group exhibited significantly higher IMF content than the LDM group (*p* < 0.05; Figure 1A), whereas shear force was significantly lower in the XJM group (*p* < 0.05; Figure 1B). No significant differences were observed between the two groups in the meat color parameters L*, a*, and b* (*p* > 0.05; Figure 1C). In addition, cooking yield was significantly lower in the XJM group than in the LDM group (*p* < 0.05; Figure 1D), whereas no significant difference in pH_45_min was observed between the two groups (*p* > 0.05; Figure 1E). These results indicate that XJM pigs were characterized by higher IMF content and lower shear force, whereas LDM pigs had a higher cooking yield.

### 3.2. Fatty Acid and Total Amino Acid Composition of the LTL Muscle in LDM and XJM Pigs

Several fatty acids showed nominal between-group differences (Table 2). However, after Benjamini–Hochberg correction for the 18 fatty-acid comparisons, only oleic acid (C18:1n-9c) remained significantly different between the groups. Oleic acid content was higher in XJM pigs than in LDM pigs (adjusted *p* = 0.037; Hedges’ g = −4.65, 95% CI: −7.98 to −1.31). C20:1, C18:2n-6, and C20:3n-3 showed nominal *p*-values < 0.05, but these differences did not remain statistically significant after multiple-testing correction (adjusted *p* = 0.152 for each comparison). No other fatty acids differed significantly after correction.

After Benjamini–Hochberg correction, XJM pigs had significantly higher contents of serine, arginine, proline, methionine, and phenylalanine than LDM pigs, whereas isoleucine and leucine contents were significantly higher in LDM pigs (adjusted *p* < 0.05; Table 3). Glycine showed a nominal between-group difference (*p* = 0.031), but this difference did not remain significant after multiple-testing correction (adjusted *p* = 0.066). No significant differences were observed for the remaining amino acids after correction.

### 3.3. Transcriptomic Analysis

**Figure 2:**
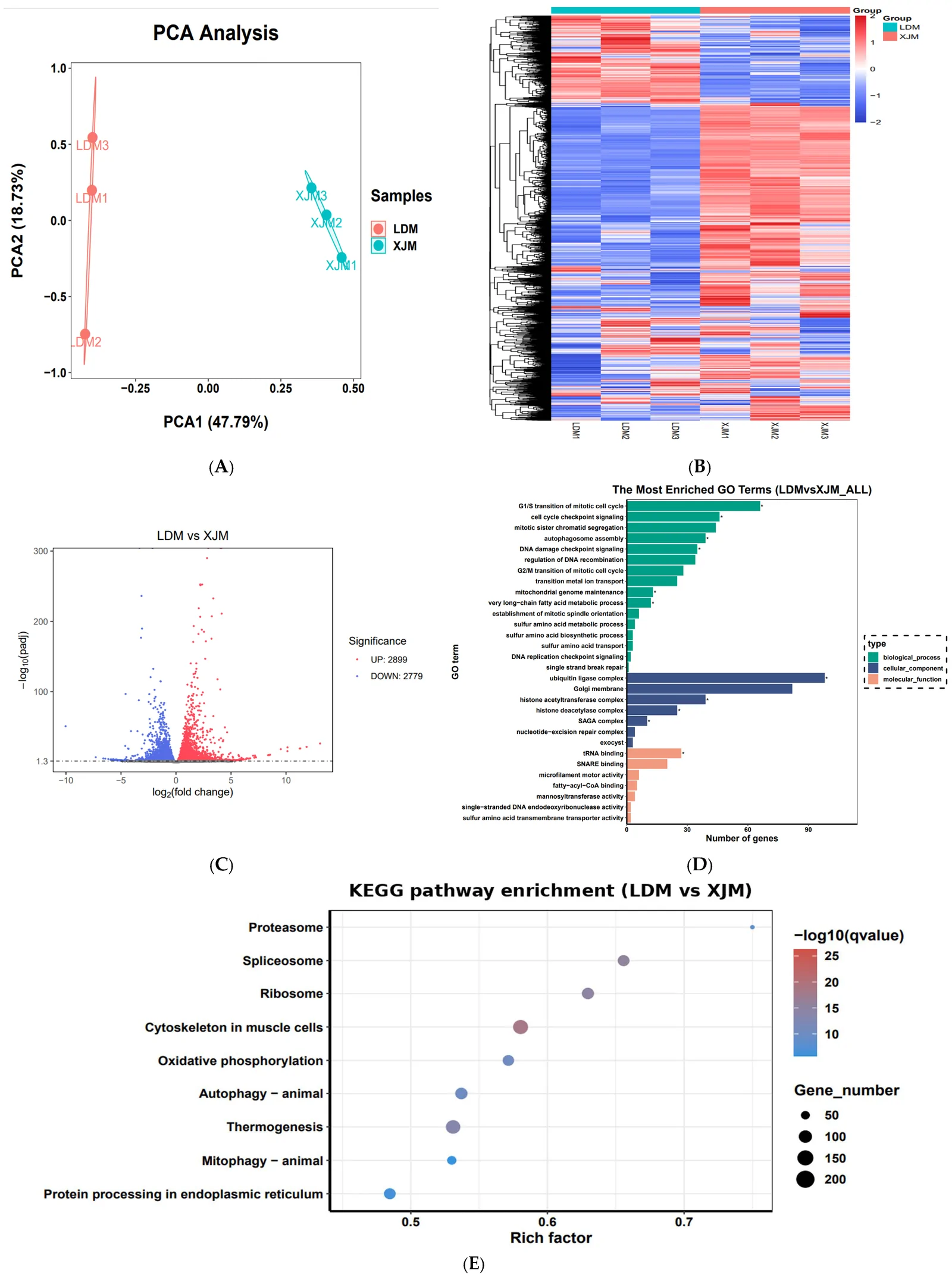
Transcriptomic analysis of the LTL muscle in Liaodan Black (LDM) and Xinjin (XJM) pigs (*n* = 3 per breed). (**A**) Principal component analysis; (**B**) hierarchical clustering heatmap of differentially expressed genes; (**C**) volcano plot of differentially expressed genes; (**D**) GO enrichment analysis of differentially expressed genes. An asterisk (*) indicates an enriched GO term; (**E**) KEGG pathway enrichment analysis of differentially expressed genes, displaying nine pathways outside the KEGG “Human Diseases” category. The corresponding 20-pathway plot, including disease-related pathways, is provided in Figure S1. In panel E, dot size represents the number of mapped differentially expressed genes, and color indicates −log_10_(q-value).

All six RNA-seq libraries showed consistently high sequencing quality, with Q30 values of 94.13–95.34%, total mapping rates of 95.99–96.93%, and uniquely mapped rates of 92.13–93.47% (Supplementary Table S2). To characterize transcriptional differences associated with meat quality variation between the LDM and XJM groups, transcriptome sequencing was performed on LTL muscle samples from both groups (Figure 2). Principal component analysis (PCA) showed clear separation between the LDM and XJM samples, indicating distinct overall gene expression profiles between the two groups (Figure 2A). Hierarchical clustering analysis of the DEGs also showed distinct expression patterns between the two groups (Figure 2B). Volcano plot analysis identified 5678 DEGs in the LDM vs. XJM comparison, including 2899 upregulated and 2779 downregulated genes (Figure 2C). GO enrichment analysis showed that, in the biological process category, DEGs were mainly enriched in G1/S transition of mitotic cell cycle, cell cycle checkpoint signaling, mitotic sister chromatid segregation, autophagosome assembly, and DNA damage checkpoint signaling. In the cellular component category, DEGs were mainly enriched in ubiquitin ligase complex, Golgi membrane, histone acetyltransferase complex, histone deacetylase complex, and SAGA complex. In the molecular function category, DEGs were mainly associated with tRNA binding, SNARE binding, microfilament motor activity, and fatty-acyl-CoA binding (Figure 2D). KEGG enrichment analysis highlighted pathways including Proteasome, Ribosome, Cytoskeleton in muscle cells, Oxidative phosphorylation, and Autophagy–animal (Figure 2E). For display, Figure 2E includes the nine pathways outside the KEGG “Human Diseases” category from the original 20-pathway plot. The original plot, including disease-related pathways, is provided in Figure S1.

#### qPCR and RNA-Seq Concordance Analysis

**Figure 3:**
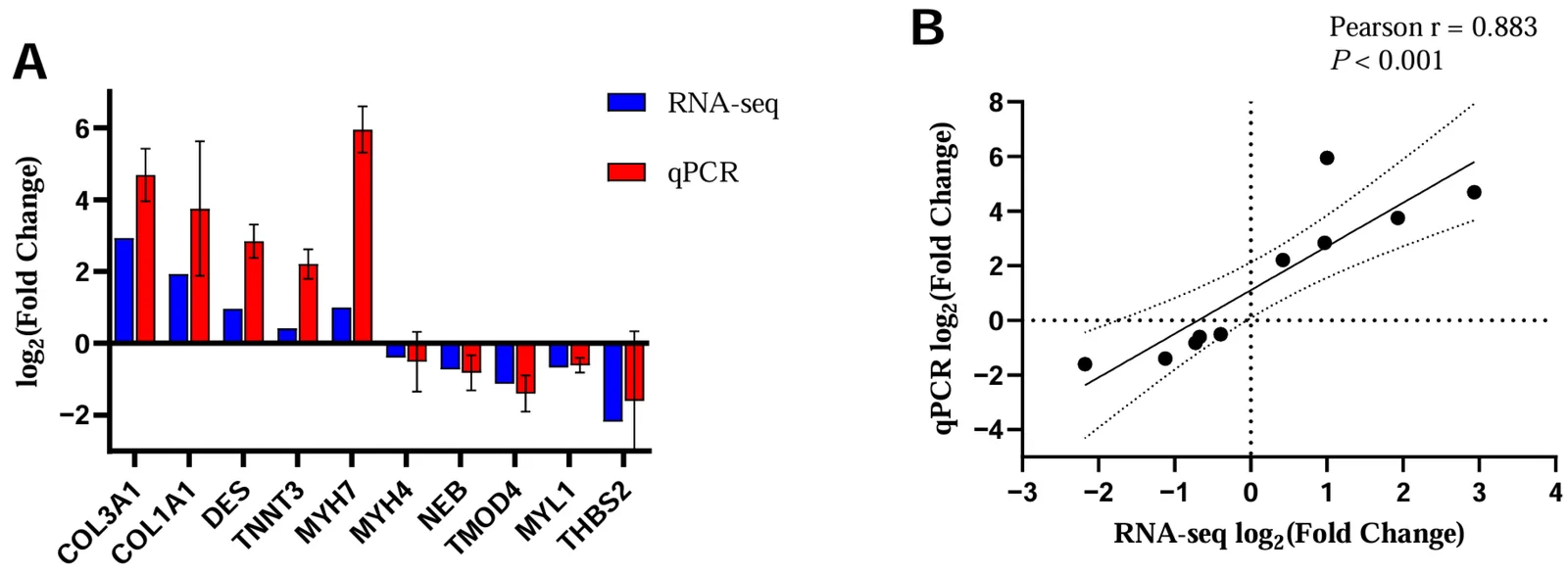
Comparison of qPCR and RNA-seq expression patterns for selected differentially expressed genes in Liaodan Black (LDM) and Xinjin (XJM) pigs. (**A**) Comparison of log_2_(fold change) values obtained by RNA-seq and qPCR for 10 selected genes. Positive log_2_(fold change) values indicate higher expression in LDM than in XJM pigs, whereas negative values indicate lower expression in LDM. qPCR error bars represent the propagated standard errors calculated from three biological replicates per breed; RNA-seq bars represent the estimated log_2_(fold change) values. (**B**) Pearson correlation between the log_2_(fold change) values obtained by RNA-seq and qPCR (r = 0.883, *p* < 0.001). In panel B, the solid line represents the fitted linear regression line, and the two flanking dotted lines indicate the 95% confidence bands. The horizontal and vertical dotted lines indicate zero log_2_(fold change) on the respective axes.

To assess the consistency of gene expression changes determined by RNA-seq, 10 DEGs were selected for qPCR analysis (Figure 3). The expression patterns determined by qPCR were consistent with those obtained by RNA-seq. COL3A1, COL1A1, DES, TNNT3, and MYH7 showed higher expression in the LDM group, whereas MYH4, NEB, TMOD4, MYL1, and THBS2 showed lower expression, consistent with the RNA-seq results (Figure 3A). Further correlation analysis of the log_2_(fold change) values obtained by the two methods revealed a significant positive correlation between qPCR and RNA-seq results (Pearson r = 0.883, *p* < 0.001; Figure 3B), indicating good agreement between the two methods.

### 3.4. Proteomic Analysis

**Figure 4:**
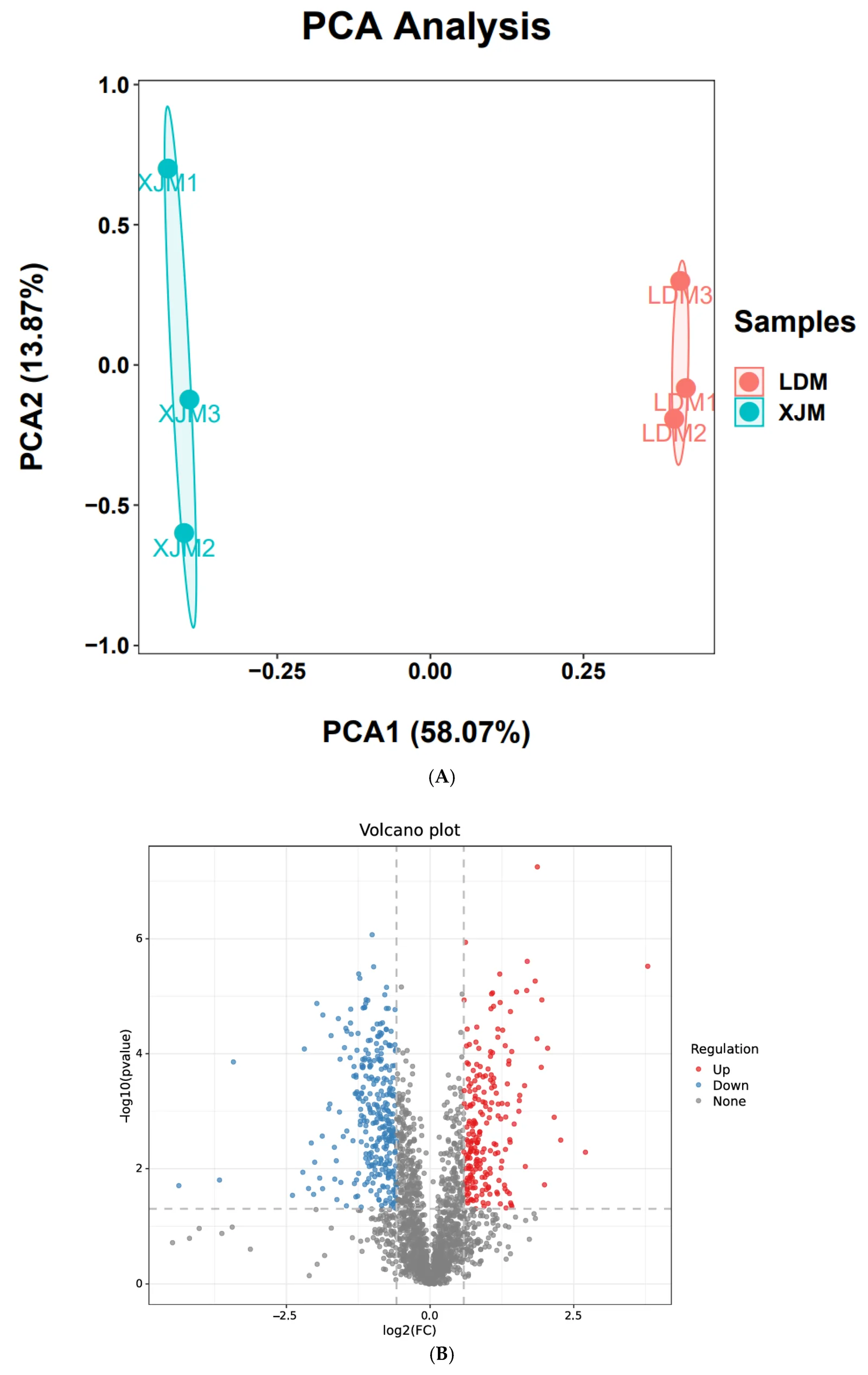

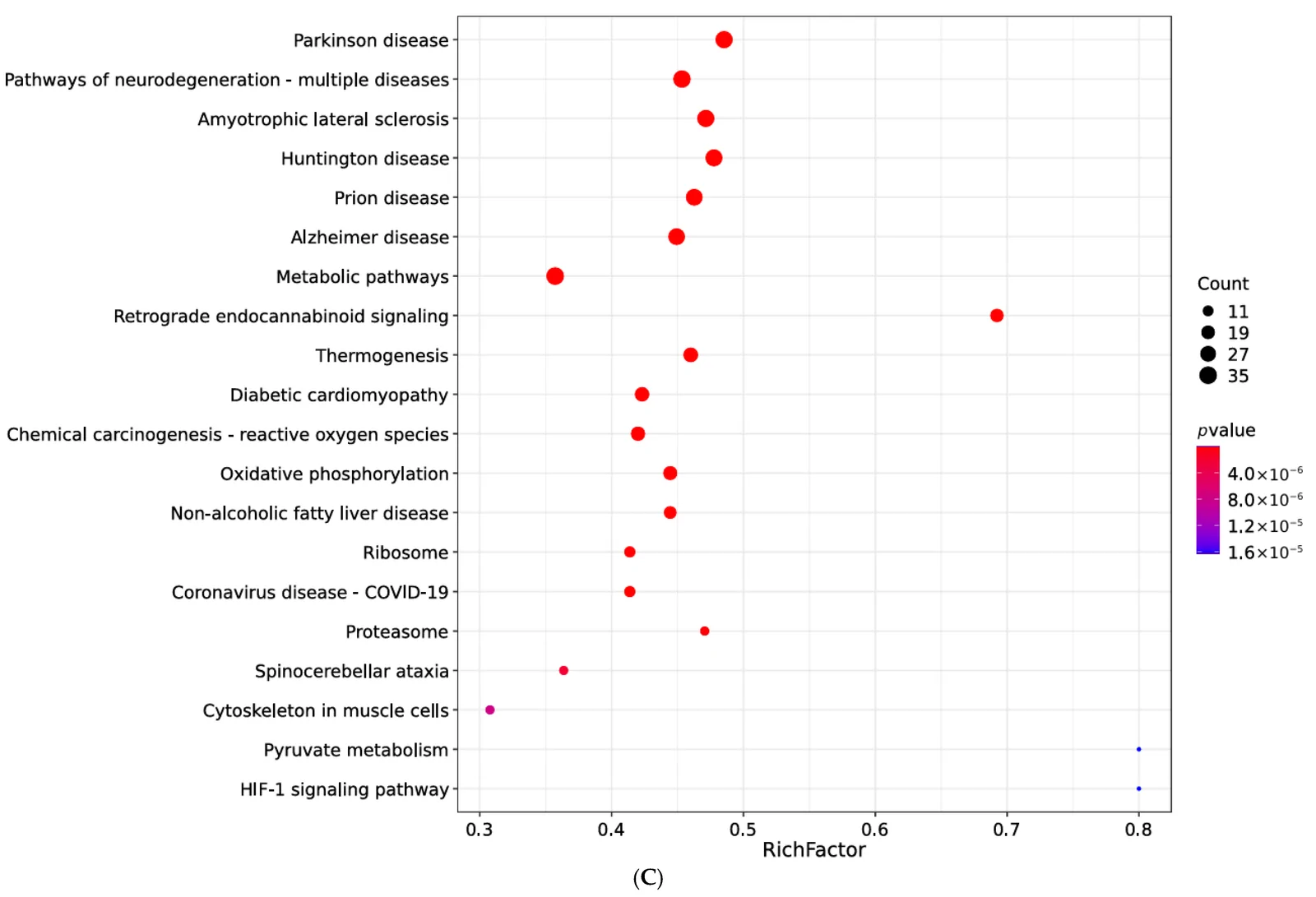
Proteomic analysis of the LTL muscle in Liaodan Black (LDM) and Xinjin (XJM) pigs (*n* = 3 per breed). (**A**) Principal component analysis; (**B**) volcano plot of differentially abundant proteins. In panel B, the horizontal dashed line indicates the nominal significance threshold (*p* = 0.05; −log_10_(p) = 1.301), while the two vertical dashed lines indicate the fold-change thresholds (log_2_FC = ±0.585, corresponding to FC = 1.5 and FC = 1/1.5); (**C**) KEGG pathway enrichment analysis of differentially abundant proteins. Dot size represents the number of mapped proteins, and dot color represents the nominal enrichment *p*-value. Statistical significance was assessed using Benjamini–Hochberg-adjusted *p*-value. Hierarchical clustering and GO enrichment analyses are presented in Figure S2.

Proteomic analysis was performed to characterize differences in LTL muscle protein abundance between LDM and XJM pigs. PCA showed clear separation between the two groups (Figure 4A). A total of 2067 proteins were identified and quantified, of which 533 were differentially abundant between the two groups, including 218 with higher abundance and 315 with lower abundance in LDM pigs than in XJM pigs (Figure 4B). KEGG enrichment analysis highlighted pathways including Oxidative phosphorylation, Ribosome, Proteasome, and Pyruvate metabolism (Figure 4C). Hierarchical clustering of the DAPs showed distinct abundance patterns between the two groups (Figure S2A). GO enrichment analyses across the biological process, cellular component, and molecular function categories are presented in Figure S2B–D. Enriched terms included mitochondrial respiratory chain complex I assembly, mitochondrial electron transport from NADH to ubiquinone, translation, and NADH dehydrogenase activity.

### 3.5. Metabolomic Analysis

**Figure 5:**
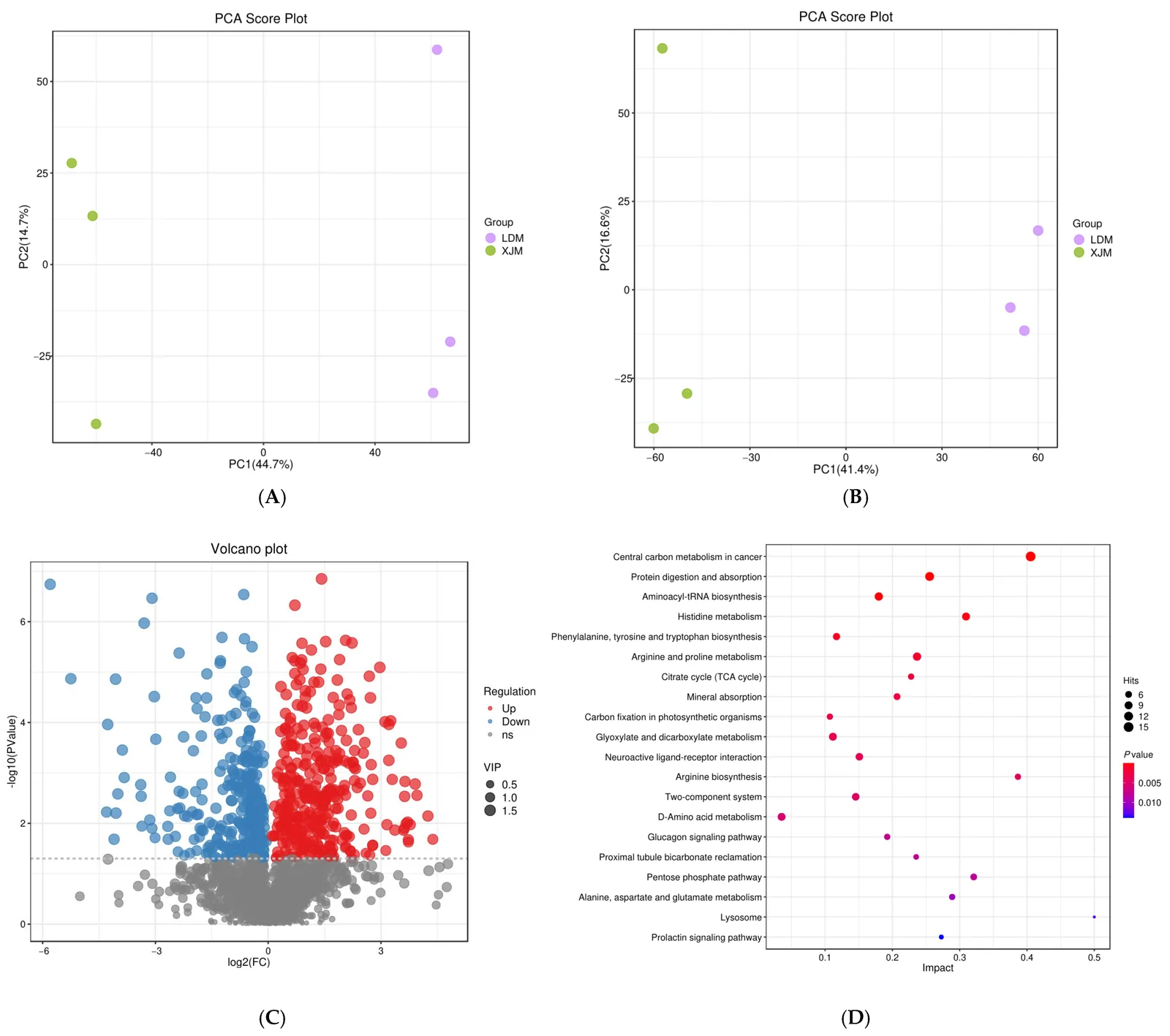
Metabolomic analysis of the LTL muscle in Liaodan Black (LDM) and Xinjin (XJM) pigs (*n* = 3 per breed). (**A**) Principal component analysis in positive-ion mode; (**B**) principal component analysis in negative-ion mode; (**C**) volcano plot of differential metabolites; (**D**) KEGG pathway enrichment analysis of differential metabolites. Dot size represents the number of mapped metabolites, and dot color represents the nominal enrichment *p*-value. Statistical significance was assessed using Benjamini–Hochberg-adjusted *p*-values. The OPLS-DA score plots used to derive VIP values for differential-metabolite screening, together with the corresponding permutation tests and hierarchical clustering analysis, are presented in Figure S3.

PCA showed separation between LDM and XJM samples in both positive- and negative-ion modes. PC1 and PC2 explained 44.7% and 14.7% of the total variance, respectively, in positive-ion mode (Figure 5A), and 41.4% and 16.6%, respectively, in negative-ion mode (Figure 5B). OPLS-DA was subsequently used to derive VIP values for differential-metabolite screening, while the OPLS-DA score plots and corresponding 100-permutation tests are presented in Figure S3A–D. Based on MS/MS spectral information and database matching, a total of 1844 metabolites were putatively annotated. Using the combined criteria of *p* < 0.05 and OPLS-DA-derived VIP > 1, a total of 622 metabolites met the differential-abundance criteria and were classified as differential metabolites, including 337 with higher relative abundance and 285 with lower relative abundance in LDM pigs than in XJM pigs (Figure 5C). KEGG enrichment analysis highlighted pathways including Aminoacyl-tRNA biosynthesis, Histidine metabolism, and Phenylalanine, tyrosine and tryptophan biosynthesis (Figure 5D). Hierarchical clustering of the differential metabolites showed distinct abundance patterns between the two groups (Figure S3E).

### 3.6. Integrated Multi-Omics Analysis

**Figure 6:**
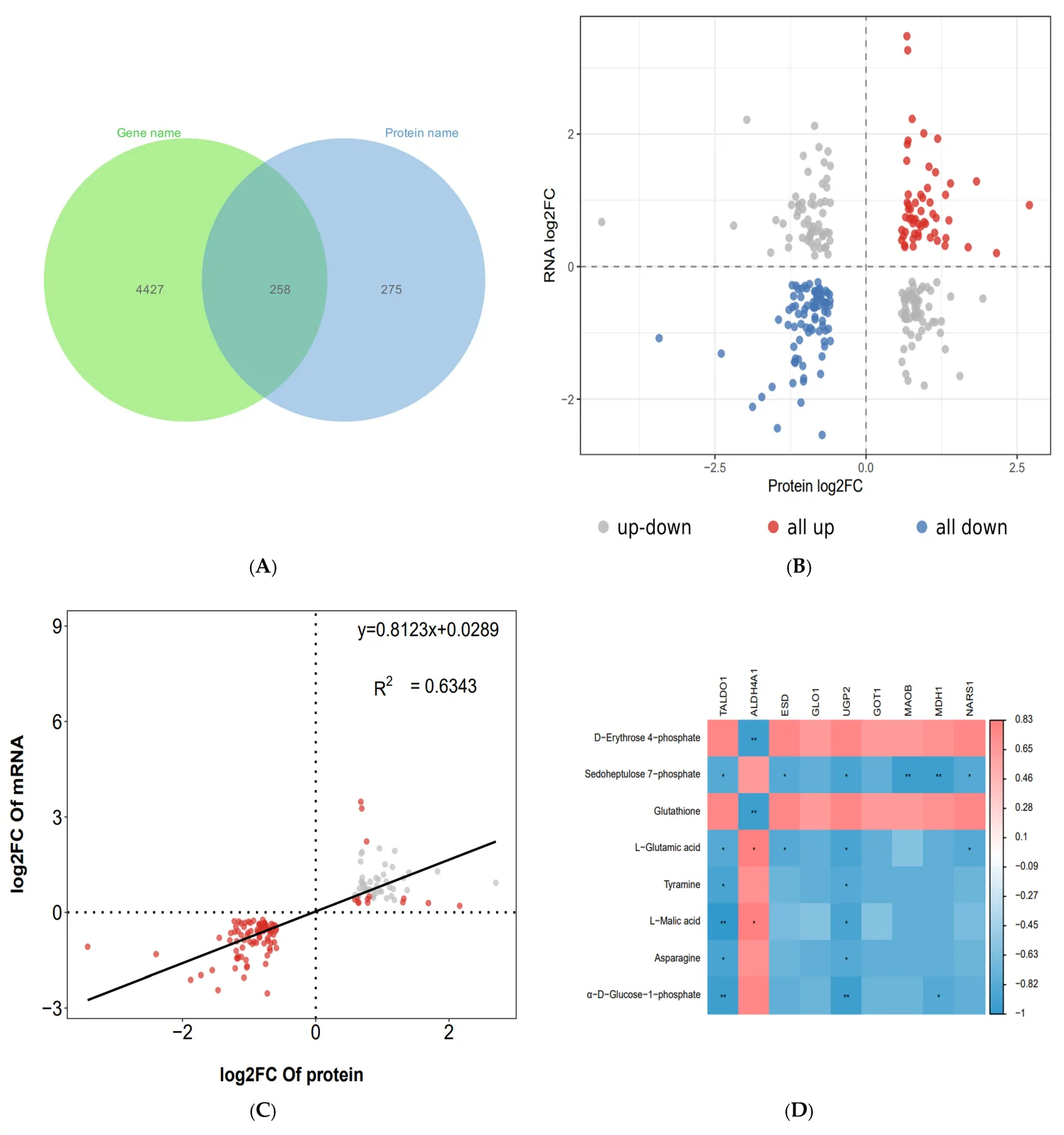

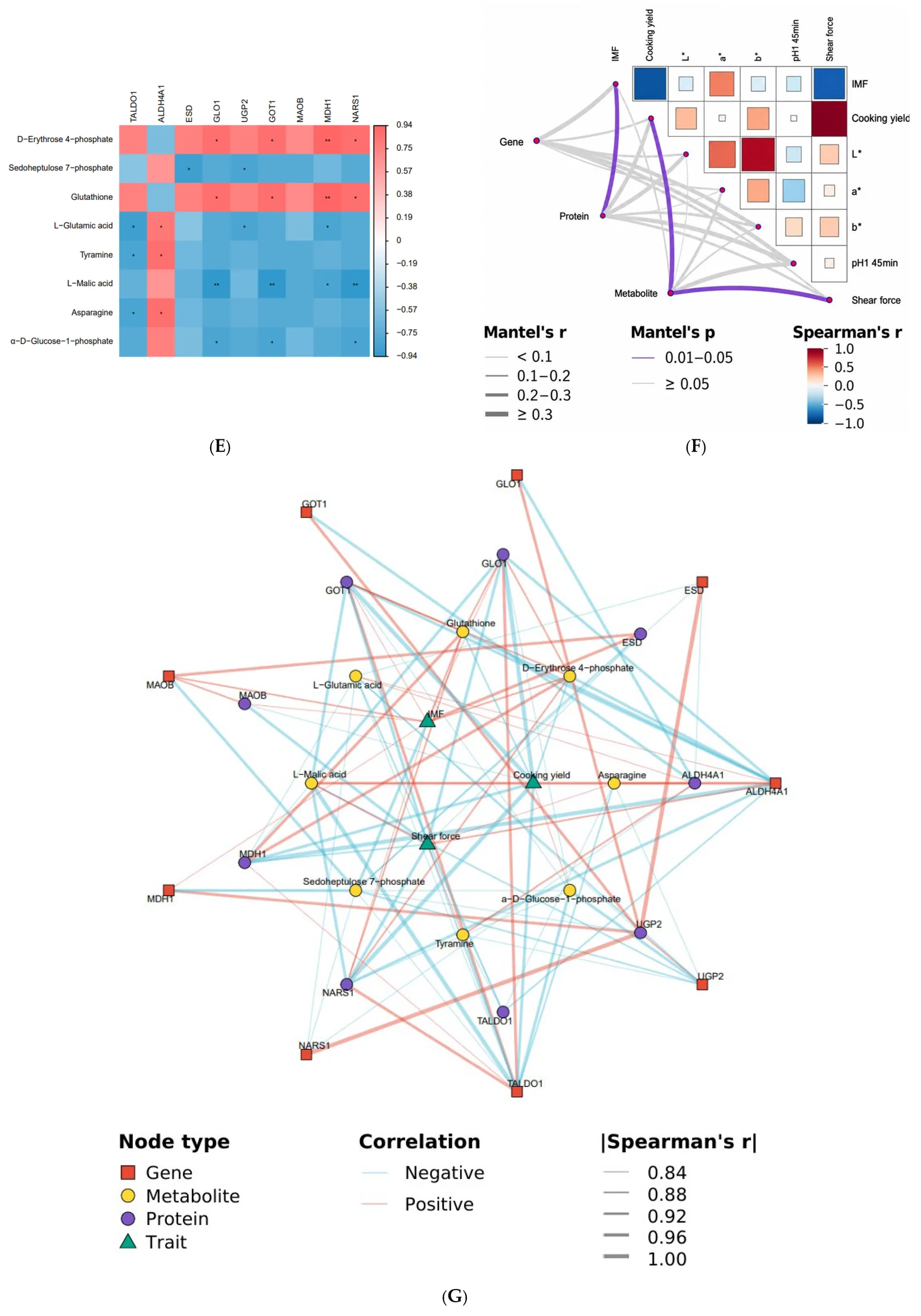
Integrated multi-omics analysis of the LTL muscle in Liaodan Black (LDM) and Xinjin (XJM) pigs (*n* = 3 per breed). (**A**) Venn diagram of differentially expressed genes and differentially abundant proteins; (**B**) quadrant plot of integrated transcriptomic and proteomic analysis; (**C**) Linear regression between protein and mRNA log_2_FC values. Each point represents one of the 139 genes showing concordant transcript- and protein-level changes. The x- and y-axes show protein and mRNA log_2_FC values, respectively. The solid line indicates the fitted regression. Red and gray points indicate statistically significant (*p* < 0.05) and nonsignificant (*p* ≥ 0.05) results, respectively; (**D**) Spearman correlations between the transcript abundances of nine candidate genes and the abundances of eight differential metabolites; (**E**) Spearman correlations between the abundances of the corresponding proteins and the same metabolites; (**F**) Mantel-test associations between molecular profiles and meat quality traits. The Gene, Protein, and Metabolite nodes represent the corresponding molecular profiles. Connecting lines indicate associations between each molecular profile and individual meat quality traits, with line width representing Mantel’s r and line color indicating the *p*-value category shown in the legend. The upper-triangular matrix displays pairwise Spearman correlations among meat quality traits, with red indicating positive correlations and blue indicating negative correlations; (**G**) Spearman correlation network among candidate molecules, differential metabolites, and meat quality traits. Red squares represent candidate genes, purple circles represent their corresponding proteins, yellow circles represent differential metabolites, and green triangles represent meat quality traits. Only correlations with |Spearman’s ρ| ≥ 0.8 and a Benjamini–Hochberg-adjusted *p*-value < 0.05 are displayed. Pink and light-blue edges indicate positive and negative correlations, respectively, and thicker edges indicate larger absolute correlation coefficients. For panels (**D**,**E**), rows represent metabolites, and columns represent candidate genes or their corresponding proteins. Cell colors indicate Spearman’s correlation coefficients according to the respective color scales, with red indicating positive correlations and blue indicating negative correlations. Asterisks denote statistical significance: * *p* < 0.05; ** *p* < 0.01.

Integrated analysis of the transcriptomic, proteomic, and metabolomic data is shown in Figure 6. After excluding DEGs without an available gene symbol, the remaining DEGs were matched with DAPs based on gene symbols, yielding 258 shared differential features detected at both the transcript and protein levels (Figure 6A). Among these, 139 showed concordant changes at the mRNA and protein levels, including 56 concordantly upregulated and 83 concordantly downregulated features (Figure 6B). Linear regression of transcript-level log_2_FC values against protein-level log_2_FC values for the 139 features preselected for concordant directions of change yielded an R^2^ of 0.63 (Figure 6C). This regression describes the relationship between between-group fold changes within the selected subset, and its R^2^ should not be interpreted as a measure of overall transcriptome–proteome concordance.

Enzyme annotations of the concordantly changed features and their links to differential metabolites identified nine candidates: TALDO1, ALDH4A1, ESD, GLO1, UGP2, GOT1, MAOB, MDH1, and NARS1. The gene–metabolite and protein–metabolite heatmaps showed broadly similar correlation patterns (Figure 6D,E). At both molecular levels, TALDO1 was positively correlated with D-erythrose 4-phosphate and negatively correlated with sedoheptulose 7-phosphate, while GLO1 was positively correlated with glutathione. GOT1 and MDH1 showed negative correlations with L-glutamic acid and L-malic acid. In contrast, ALDH4A1 showed positive correlations with these two metabolites. The significance of individual correlations is indicated in Figure 6D,E.

Mantel tests showed that the candidate-protein profile was associated with IMF content, whereas the differential-metabolite profile was associated with cooking yield and shear force (*p* < 0.05; Figure 6F). None of the tested associations between the candidate-gene profile and meat-quality traits reached *p* < 0.05.

After Benjamini–Hochberg correction for multiple testing, the Spearman correlation network retained 40 associations satisfying |Spearman’s ρ| ≥ 0.8 and an adjusted *p*-value < 0.05 (Figure 6G). GOT1 and GLO1 protein abundances were negatively correlated with both shear force and cooking yield, whereas MDH1 protein abundance was negatively correlated with cooking yield. ESD protein abundance was positively correlated with IMF content. Among the metabolites, sedoheptulose 7-phosphate was negatively correlated with IMF content, whereas L-malic acid was positively correlated with cooking yield.

## 4. Discussion

Muscle structure and intramuscular lipid deposition contribute to meat tenderness and eating quality [12,13,14,15]. The lower shear force observed in XJM pigs is consistent with the association between higher IMF content and improved pork tenderness reported previously. However, XJM pigs also had a significantly lower cooking yield than LDM pigs, indicating reduced mass retention during cooking. This combination of higher IMF content and lower cooking yield contrasts with the higher IMF content and lower cooking loss reported by Choi et al. [16] for Duroc pigs compared with Landrace × Yorkshire × Duroc (LYD) crossbred pigs.

The contribution of intramuscular lipids to pork quality also depends on their fatty acid composition [17,18,19]. In the present study, the higher IMF content of XJM pigs was accompanied by a significantly higher oleic acid (C18:1n-9c) content after correction for multiple testing. Although linoleic acid (C18:2n-6) showed a lower mean value in XJM pigs, this difference did not remain significant after Benjamini–Hochberg correction. Kumar et al. [4] reported that commercial Large White × Landrace pigs had higher contents of both oleic and linoleic acids than Songliao Black pigs. The present results therefore provide stronger statistical support for a difference in oleic acid than for the other fatty acids examined. Fatty acids are important precursors of meat flavor, and their oxidation contributes to the formation of volatile compounds [20]. The higher oleic acid content in XJM pigs may therefore be relevant to flavor development. Navarro et al. [21] reported higher consumer liking scores for pork enriched in oleic acid through dietary manipulation. However, the sensory consequences of the fatty acid composition observed here were not evaluated. Accordingly, the present fatty-acid data primarily support a difference in oleic acid between the two groups rather than broad differences across multiple fatty-acid classes. MUFA and PUFA are also associated with lipid metabolism, cardiovascular health, glycemic regulation, and metabolic homeostasis [22,23,24]. Therefore, the observed fatty acid differences reflect distinct lipid nutritional characteristics between the two groups.

After correction for multiple testing, total amino acid analysis continued to show contrasting patterns among indispensable amino acids: methionine and phenylalanine contents were higher in XJM pigs, whereas leucine and isoleucine contents were higher in LDM pigs. By comparison, Kumar et al. [4] reported no significant differences in amino acid composition between Songliao Black pigs and commercial Large White × Landrace pigs. Protein nutritional quality depends on both indispensable amino acid composition and digestibility [25,26]. Given the opposing differences in individual indispensable amino acids and the absence of digestibility measurements, the present results do not establish superior protein nutritional quality for either group.

Transcriptomic studies have identified candidate genes associated with pork quality and breed-specific meat quality characteristics [27]. In Iberian pigs, 200 DEGs were identified, with IGF1, VGLL3, and PPARG highlighted as candidates associated with meat tenderness [28]. Similarly, integrated multi-omics analysis of commercial crossbred and local pigs identified PPARG, SREBF1, and ACACA among candidate genes associated with IMF deposition [2]. In the present study, transcriptomic analysis revealed that the major molecular differences between LDM and XJM pigs involved muscle structural organization and extracellular matrix-related processes. Among these genes, COL1A1 and COL1A2, which encode the α1 and α2 chains of type I collagen, respectively [29], showed higher expression in LDM pigs than in XJM pigs. This transcriptional pattern was consistent with the higher shear force observed in LDM pigs, suggesting that variation in extracellular matrix-associated gene expression observed between the sampled groups may contribute to the difference in muscle texture. However, because collagen content, solubility, cross-linking, and muscle histological characteristics were not measured, the relationship between collagen-related transcriptional variation and meat tenderness requires further validation.

In addition to these collagen-related transcriptional findings, both transcriptomic and proteomic analyses showed significant enrichment of the Oxidative phosphorylation pathway. Proteomic GO analysis further highlighted mitochondrial respiratory chain complex I assembly, mitochondrial electron transport (NADH to ubiquinone), and NADH dehydrogenase activity. Consistently, NDUFV1, NDUFV2, NDUFS8, NDUFS6, NDUFS5, NDUFC2, NDUFA2, and several NDUFB family members were less abundant in LDM than in XJM pigs. These proteins are components of mitochondrial respiratory chain Complex I, suggesting differences in mitochondrial protein composition and energy-metabolism-related processes between the sampled groups. As mitochondrial energy metabolism is closely associated with postmortem ATP depletion and metabolic transition in muscle [30], these differences may contribute to distinct postmortem metabolic characteristics between the two groups. However, because Complex I activity, mitochondrial respiration, and postmortem ATP dynamics were not measured, functional consequences cannot be established.

Proteomic KEGG analysis also showed enrichment of Ribosome, Proteasome, and Pyruvate metabolism pathways. Ribosome biogenesis is associated with skeletal muscle growth and fiber phenotype [31], whereas the ubiquitin–proteasome system contributes to muscle protein turnover, postmortem myofibrillar degradation, and tenderness development [32,33]. Pyruvate metabolism links glycolysis with lactate production and mitochondrial oxidation and is closely associated with postmortem pH decline [34]. The enrichment of Ribosome and Proteasome in both the transcriptomic and proteomic analyses places protein turnover among the processes potentially related to the shear-force difference, although the direction of postmortem proteolysis was not determined. Pyruvate metabolism was enriched at the protein level, but pH_45_min did not differ significantly between LDM and XJM pigs. The cooking-yield difference therefore cannot be attributed to a demonstrated difference in early postmortem pH.

Metabolomic KEGG enrichment analysis showed that the differential metabolites between the two groups were mainly associated with amino acid metabolism and central carbon metabolism. Further examination of amino acid-related metabolites revealed higher relative abundances of L-arginine, L-proline, and L-glutamic acid in the LDM group than in the XJM group. This pattern differed from the acid hydrolysis-based amino acid composition analysis, in which arginine and proline contents were higher in the XJM group; however, these findings are not necessarily contradictory. Acid hydrolysis reflects the overall amino acid composition of muscle proteins, whereas untargeted metabolomics primarily captures the relative abundance of extractable low-molecular-weight metabolites in muscle tissue. Thus, the two approaches characterize different aspects of amino acid profiles, and differences in their directional trends can occur [35]. From a metabolic perspective, arginine participates in nitric oxide synthesis, the urea cycle, and polyamine metabolism [36]. Proline and its hydroxylated derivative hydroxyproline are important components of collagen and are closely associated with connective tissue metabolism [37]. Together with the upregulation of collagen- and extracellular matrix-related genes such as COL1A1 and COL1A2 in the LDM group, the higher relative abundance of L-proline in LDM pigs suggests a potential association between amino acid metabolism and extracellular matrix-related molecular characteristics observed between the two groups. These metabolomic findings complement the muscle structure-related differences observed at the transcriptomic level; however, the specific relationships between these molecular changes remain to be further validated.

From an agriculture-to-food perspective, integrating meat-quality phenotypes with transcriptomic, proteomic, and metabolomic information provides a means to connect regional pig genetic resources with measurable pork-quality outcomes. Previous studies have also provided useful insights into the meat quality characteristics of local pig breeds [3]. By jointly analyzing muscle phenotypes, metabolites, gene expression profiles, and/or genetic loci, several studies have identified molecular features associated with the development of meat quality traits [38]. However, important limitations remain. Many previous studies have focused primarily on pairwise associations between metabolites and meat quality traits or between genes and meat quality traits, or have examined individual characteristics such as muscle fiber type or IMF content. Consequently, systematic relationships between multidimensional molecular features and multiple meat quality traits have not yet been fully established. The coordinated relationships among different molecular layers involved in meat quality development therefore remain incompletely understood, and the molecular basis underlying breed-related differences in meat quality phenotypes requires further investigation [39].

Among the shared differential features, 53.9% changed in the same direction at the mRNA and protein levels. This partial concordance indicates that the direction of transcriptional changes did not consistently predict the corresponding protein-level differences. Integration with metabolites therefore focused on enzymatic candidates with concordant changes at both molecular levels. The evidence supporting these candidates differed in its biological specificity. Published adipocyte experiments provide functional context for the metabolic role of GOT1 [40], while the TALDO1 findings connect transcript and protein abundance with specific sugar-phosphate intermediates.

The GOT1–MDH1 associations suggest a potential metabolic connection between amino-acid utilization and muscle metabolic characteristics. Yang et al. [40] demonstrated that overexpression of porcine GOT1 in mouse 3T3-L1 cells increased lipid accumulation, NADPH content, and the expression of MDH1 and ME1, whereas GOT1 disruption produced the opposite effects. In the present study, GOT1 and MDH1 were also associated with glutamate- and malate-related metabolic variation at the transcript and protein levels. In the BH-corrected correlation network, GOT1 protein abundance remained negatively associated with shear force and cooking yield. These observations support GOT1 as a candidate linking metabolic variation with meat-quality characteristics, although the present correlations do not establish a causal relationship. TALDO1 transcript and protein levels showed positive correlations with D-erythrose 4-phosphate and negative correlations with sedoheptulose 7-phosphate. Of these four correlations, only the transcript-level association with sedoheptulose 7-phosphate reached *p* < 0.05. These findings link TALDO1 expression and protein abundance with variation in sugar-phosphate intermediates, although they do not establish increased pathway activity. In a metabolomic analysis of Duroc × Pietrain pigs, Welzenbach et al. [41] identified associations of glycolytic and pentose phosphate pathway metabolites with postmortem pH and meat color, providing additional context for the potential relevance of sugar-phosphate metabolism to meat-quality variation. After correction for multiple testing, GOT1 and GLO1 protein abundances remained negatively associated with both shear force and cooking yield, while MDH1 protein abundance was negatively associated with cooking yield. L-malic acid was positively associated with cooking yield, whereas ESD protein abundance and sedoheptulose 7-phosphate showed positive and negative associations with IMF content, respectively. These corrected associations indicate that the molecular features related to tenderness, cooking performance, and intramuscular lipid deposition were not identical, supporting the interpretation of these meat-quality characteristics as related but distinct components of the observed phenotype. The molecular analyses were based on three pigs per breed, providing limited information about within-breed biological variability. In addition, complete pedigree records were not available for the selected animals, and the degree of relatedness among the three pigs within each breed could therefore not be determined. Potential familial relatedness among the sampled animals cannot be excluded and may further limit their representation of the broader within-breed population. Future studies should include larger independently sampled cohorts with documented pedigree information and, where possible, animals representing multiple families within each breed. The same six pigs supplied all three omics datasets, and qPCR was performed using the same muscle samples. Concordance among these assays therefore does not constitute validation in an independent animal cohort. Each breed was also housed in a single pen, resulting in complete confounding between breed and pen-associated environmental effects. Consequently, the observed differences cannot be attributed exclusively to breed, and independent replication of pens within breed will be required to distinguish genetic breed effects from housing-related environmental variation. Therefore, the observed molecular and phenotypic differences should be interpreted as differences between the sampled LDM and XJM groups under the present management conditions rather than definitive estimates of genetic breed effects. Furthermore, the correlation network was constructed using the same animals from which the differential features were selected, and the observed associations may partly reflect separation between the two groups. Accordingly, the molecular findings and correlation network are interpreted as exploratory results for candidate prioritization. The reproducibility of the priority findings requires assessment in a larger, independently sampled cohort, while proposed functional roles require direct experimental investigation.

## 5. Conclusions

This exploratory study characterized meat quality and molecular differences in three LDM and three XJM pigs. XJM pigs exhibited higher IMF and oleic acid contents and lower shear force, whereas LDM pigs showed higher cooking yield and higher transcript levels of extracellular matrix-related genes. Multi-omics integration identified candidate molecular features associated with lipid deposition, extracellular matrix-related processes, mitochondrial protein composition, and central carbon metabolism. Among these findings, GOT1 and GLO1 protein abundances were negatively associated with shear force and cooking yield in the multiple-testing-corrected exploratory network, while TALDO1 transcript and protein abundances were associated with sugar-phosphate intermediates. These associations provide hypotheses for further investigation; their reproducibility and relevance to meat quality require evaluation in larger, independent cohorts.

## Figures and Tables

**Table 1:** Primer sequences and amplicon sizes used for qPCR analysis.

Gene Symbol	Official Gene Name	GenBank/RefSeq Accession No.	Primer Sequence (5′–3′)	Amplicon Size (bp)
*GAPDH*	glyceraldehyde-3-phosphate dehydrogenase	NM_001206359.1	F: GCACAGTCAAGGCGGAGA R: GGCAGAAGGGGCAGAGAT	206
*COL3A1*	collagen type III alpha 1 chain	NM_001243297.1	F: TGCTCTACTTCATCCCACTGTTATT R: CCGATTCCTCCTCCTGCTACT	474
*COL1A1*	collagen type I alpha 1 chain	NM_001444319.1	F: CTGGTATTGCTGGTGCTCCTGG R: GGCTCCTCGCTTTCCTTCTTCT	206
*DES*	desmin	NM_001001535.1	F: GCCGAAGAGTGGTACAAGTCAAA R: TGCCTCATCAGGGAATCGTTAG	170
*TNNT3*	troponin T3, fast skeletal type	NM_001001863.2	F: CAGTGAGGACAAGCTGAGGGAC R: TTCCACCTTCCGCCAACC	201
*MYH7*	myosin heavy chain 7	NM_213855.2	F: AGCAGAATCCACCCAAGT R: CGGCAATGACAGCAAAGT	366
*MYH4*	myosin, heavy chain 4, skeletal muscle	NM_001123141.1	F: AAGACTGTGAACACGAAGC R: GATGAATTTACCAAAGCGA	201
*NEB*	nebulin	XM_021075466.2	F: AAGTTACCGACTCTCCTGTT R: TCTTAGCTTTGGTCTTCTCC	204
*TMOD4*	tropomodulin 4	NM_001243294.1	F: ACAGGCGAGAAGAAGGGG R: GTTTGTGGGATTTGGGGG	282
*MYL1*	myosin light chain 1	NM_214374.2	F: AACCCCAGCAATGAAGAGA R: TCAAAGACACGCAGACCCT	131
*THBS2*	thrombospondin 2	XM_013992423.3	F: TGCGACGTGACCAGCAACAC R: CGGAGCAGGAGGACCAAGGA	130

Note: F, forward primer; R, reverse primer. Accession numbers identify representative Sus scrofa RefSeq transcripts containing the primer-binding sites.

**Table 2:** Fatty acid composition of the LTL muscle in Liaodan Black and Xinjin pigs.

Fatty Acid	Category	LDM (μg/g)	XJM (μg/g)	*p*-Value	Adjusted *p*-Value	Hedges’ g (95% CI)
C14:0	SFA	53.00 ± 11.55	51.89 ± 4.81	0.885	0.885	0.10 (−1.18, 1.37)
C16:0	SFA	1381.66 ± 204.88	1264.89 ± 274.51	0.587	0.704	0.38 (−0.94, 1.67)
C18:0	SFA	852.83 ± 125.03	659.76 ± 130.10	0.137	0.274	1.21 (−0.35, 2.67)
C16:1	MUFA	127.09 ± 42.83	189.59 ± 32.56	0.115	0.258	−1.31 (−2.81, 0.29)
C18:1n-9c	MUFA	1447.35 ± 156.20	2300.97 ± 135.90	0.002	0.037	−4.65 (−7.98, −1.31)
C18:1n-7	MUFA	193.39 ± 53.41	266.27 ± 15.72	0.086	0.221	−1.48 (−3.04, 0.19)
C20:1	MUFA	24.83 ± 4.75	38.76 ± 5.94	0.034	0.152	−2.07 (−3.90, −0.14)
C18:2n-6	n-6 PUFA	1000.16 ± 167.60	553.87 ± 150.63	0.027	0.152	2.23 (0.23, 4.15)
C18:3n-6	n-6 PUFA	3.54 ± 0.53	2.79 ± 0.18	0.084	0.221	1.49 (−0.18, 3.06)
C20:3n-6	n-6 PUFA	20.23 ± 7.25	15.61 ± 3.44	0.375	0.533	0.65 (−0.73, 1.96)
C20:4n-6	n-6 PUFA	177.32 ± 46.00	151.81 ± 38.97	0.504	0.648	0.48 (−0.87, 1.77)
C22:4n-6	n-6 PUFA	21.48 ± 1.80	19.40 ± 3.24	0.385	0.533	0.63 (−0.74, 1.95)
C22:5n-6	n-6 PUFA	3.65 ± 0.63	2.86 ± 0.62	0.197	0.323	1.01 (−0.48, 2.40)
C18:3n-3	n-3 PUFA	10.57 ± 2.64	10.15 ± 2.41	0.851	0.885	0.13 (−1.16, 1.40)
C20:3n-3	n-3 PUFA	3.95 ± 0.33	2.54 ± 0.69	0.034	0.152	2.07 (0.14, 3.90)
C20:5n-3	n-3 PUFA	3.97 ± 0.75	2.79 ± 0.42	0.075	0.221	1.56 (−0.14, 3.16)
C22:5n-3	n-3 PUFA	17.82 ± 3.46	13.37 ± 2.65	0.152	0.274	1.15 (−0.39, 2.59)
C22:6n-3	n-3 PUFA	6.09 ± 1.89	5.73 ± 0.60	0.771	0.868	0.20 (−1.09, 1.47)

Note: Values are presented as the mean ± SD (*n* = 3 per breed). Fatty acid contents are expressed as micrograms per gram of fresh muscle tissue (μg/g fresh weight). SFA, saturated fatty acids; MUFA, monounsaturated fatty acids; PUFA, polyunsaturated fatty acids. Nominal *p*-values were obtained using two-sided independent-samples Student’s *t*-tests and were adjusted for the 18 fatty-acid comparisons using the Benjamini–Hochberg correction procedure. Hedges’ g is presented with its 95% confidence interval (CI); positive values indicate higher values in LDM, whereas negative values indicate higher values in XJM. The 95% CIs for Hedges’ g were not adjusted for multiplicity. Statistical significance was determined using an adjusted *p*-value < 0.05.

**Table 3:** Total amino acid composition of the LTL muscle in Liaodan Black and Xinjin pigs.

Parameter	LDM (mg/g)	XJM (mg/g)	*p*-Value	Adjusted *p*-Value	Hedges’ g (95% CI)
Aspartic acid	7.711 ± 0.081	7.484 ± 0.451	0.440	0.467	0.56 (−0.80, 1.86)
Glutamic acid	11.745 ± 0.102	12.192 ± 0.671	0.318	0.409	−0.74 (−2.07, 0.67)
Serine	3.264 ± 0.078	3.881 ± 0.217	0.010	0.024	−3.01 (−5.35, −0.60)
Arginine	9.853 ± 0.178	11.294 ± 0.181	<0.001	0.003	−6.41 (−10.87, −2.00)
Glycine	2.550 ± 0.040	2.818 ± 0.137	0.031	0.066	−2.13 (−3.99, −0.17)
Threonine	5.908 ± 0.123	6.472 ± 0.383	0.072	0.123	−1.58 (−3.19, 0.13)
Proline	6.836 ± 0.326	7.738 ± 0.003	0.009	0.024	−3.13 (−5.53, −0.65)
Alanine	6.658 ± 0.126	6.733 ± 0.361	0.753	0.753	−0.22 (−1.49, 1.08)
Valine	6.436 ± 0.030	6.679 ± 0.369	0.317	0.409	−0.74 (−2.08, 0.66)
Methionine	2.309 ± 0.032	2.982 ± 0.187	0.004	0.015	−3.99 (−6.92, −1.03)
Cysteine	5.383 ± 0.065	5.903 ± 0.330	0.055	0.104	−1.75 (−3.42, 0.04)
Isoleucine	12.049 ± 0.045	8.705 ± 0.520	<0.001	0.003	7.23 (2.31, 12.21)
Leucine	0.580 ± 0.028	0.343 ± 0.023	<0.001	0.003	7.33 (2.35, 12.38)
Phenylalanine	6.147 ± 0.086	7.017 ± 0.293	0.008	0.024	−3.22 (−5.68, −0.69)
Histidine	3.351 ± 0.235	3.162 ± 0.227	0.372	0.421	0.65 (−0.73, 1.97)
Lysine	5.890 ± 0.132	5.461 ± 0.576	0.276	0.409	0.82 (−0.61, 2.17)
Tyrosine	4.605 ± 0.238	4.906 ± 0.414	0.336	0.409	−0.71 (−2.04, 0.69)

Note: Values are presented as the mean ± SD (*n* = 3 per breed). Amino acid contents are expressed as milligrams per gram of fresh muscle tissue (mg/g fresh weight). Nominal *p*-values were obtained using two-sided independent-samples Student’s *t*-tests and were adjusted for the 17 amino-acid comparisons using the Benjamini–Hochberg correction procedure. Hedges’ g is presented with its 95% confidence interval (CI); positive values indicate higher values in LDM, whereas negative values indicate higher values in XJM. The 95% CIs for Hedges’ g were not adjusted for multiplicity. Statistical significance was determined using an adjusted *p*-value < 0.05.

## Data Availability

The mass spectrometry proteomics data have been deposited to the ProteomeXchange Consortium via the PRIDE partner repository with the dataset identifier PXD084581. During peer review, the PRIDE dataset is private, and reviewer access credentials have been provided separately through the journal submission system. The raw LC–MS metabolomics data, associated sample and assay metadata, and metabolite annotation tables have been deposited in the MetaboLights repository under study accession MTBLS15873 (https://www.ebi.ac.uk/metabolights/MTBLS15873 (accessed on 30 September 2026)). The MetaboLights dataset is currently private and can be accessed by the editors and reviewers using the reviewer link and access token provided separately through the journal submission system. Both datasets will be made publicly available upon publication. Additional results are provided in the article and its Supplementary Materials.

## Supplementary Materials

The following supporting information can be downloaded at https://www.mdpi.com/article/10.3390/foods15203656/s1: Figure S1: KEGG pathway enrichment analysis of differentially expressed genes, including disease-related pathways; Figure S2: Hierarchical clustering and GO enrichment analyses of differentially abundant proteins; Figure S3: OPLS-DA score plots, permutation tests, and hierarchical clustering analysis of differential metabolites; Figure S4: DESeq2 dispersion estimates for RNA-seq differential-expression analysis; Table S1: Individual measurements of meat quality traits, fatty acid composition, and total amino acid composition; Table S2: RNA-seq sequencing quality and alignment statistics; Table S3A: Individual amino acid concentrations and corresponding laboratory sample identifiers; Table S3B: Statistical verification of the amino acid composition results presented in Table 3; Table S4: Relative LC–MS acquisition order of biological samples and extraction blanks.
