# Supplementary material for: Multi-Omics Integration Reveals Molecular Signatures Associated with Pork Quality Differences Between Liaodan Black and Xinjin Pigs
Source: Foods. 2026 Oct 15;15(20):3656. doi: 10.3390/foods15203656 (PMC13703621; doi:10.3390/foods15203656)
Supplement: Supplementary file 1 [file foods-15-03656-s001.zip › foods-4607398-supplementary/Supplementary_Materials_Organized/Figures/Figure_S1_Transcriptomic_KEGG_20_Pathways.pdf]

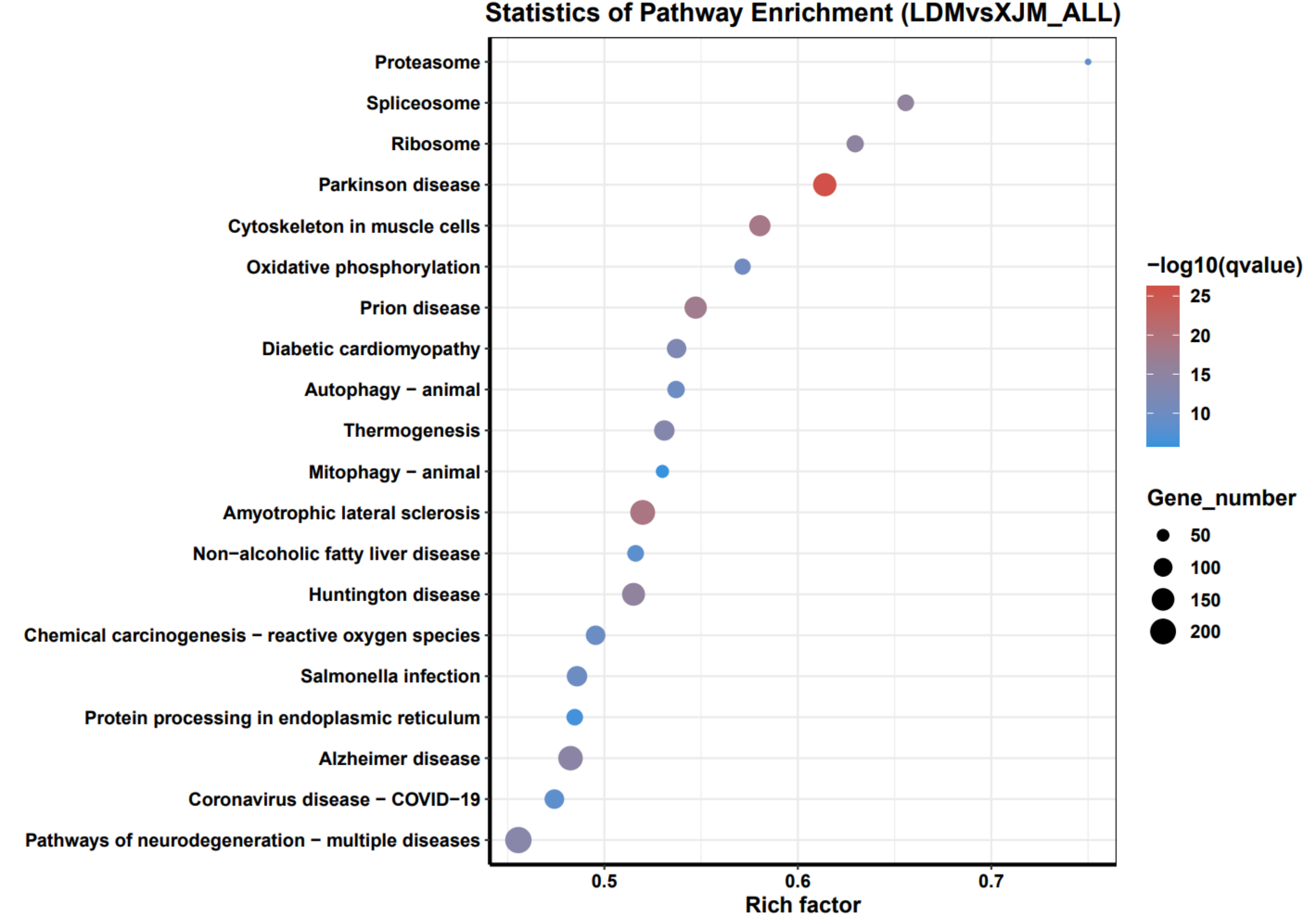

Statistics of Pathway Enrichment (LDMvsXJM_ALL)
Proteasome
Spliceosome
Ribosome
Parkinson disease
Cytoskeleton in muscle cells
Oxidative phosphorylation
Prion disease
Diabetic cardiomyopathy
Autophagy − animal
Thermogenesis
Mitophagy − animal
Amyotrophic lateral sclerosis
Non−alcoholic fatty liver disease
Huntington disease
Chemical carcinogenesis − reactive oxygen species
Salmonella infection
Protein processing in endoplasmic reticulum
Alzheimer disease
Coronavirus disease − COVID−19
Pathways of neurodegeneration − multiple diseases
0.5
0.6
0.7
Rich factor
−log10(qvalue)
25
20
15
10
Gene_number
50
100
150
200
