# Supplementary material for: Multi-Omics Integration Reveals Molecular Signatures Associated with Pork Quality Differences Between Liaodan Black and Xinjin Pigs
Source: Foods. 2026 Oct 15;15(20):3656. doi: 10.3390/foods15203656 (PMC13703621; doi:10.3390/foods15203656)
Supplement: Supplementary file 1 [file foods-15-03656-s001.zip › foods-4607398-supplementary/Supplementary_Materials_Organized/Figures/Figure_S2B_Proteomic_GO_Biological_Process.pdf]

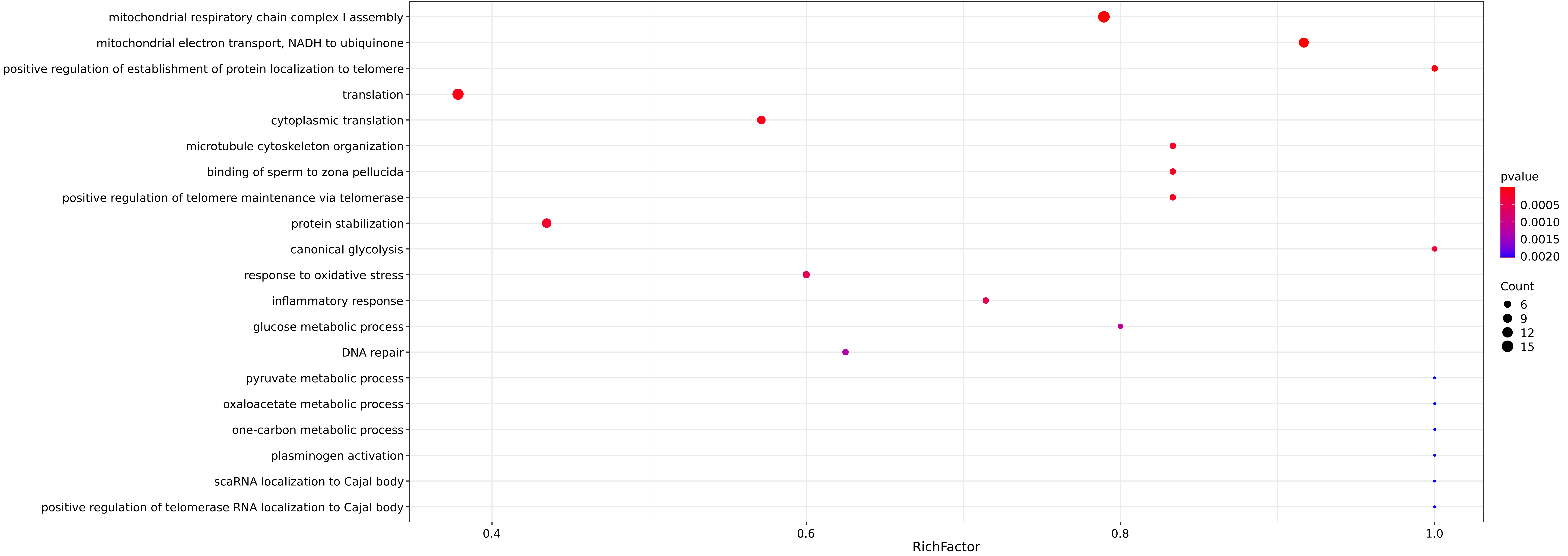

mitochondrial respiratory chain complex I assembly
mitochondrial electron transport, NADH to ubiquinone
positive regulation of establishment of protein localization to telomere
translation
cytoplasmic translation
microtubule cytoskeleton organization
binding of sperm to zona pellucida
positive regulation of telomere maintenance via telomerase
protein stabilization
canonical glycolysis
response to oxidative stress
inflammatory response
glucose metabolic process
DNA repair
pyruvate metabolic process
oxaloacetate metabolic process
one-carbon metabolic process
plasminogen activation
scaRNA localization to Cajal body
positive regulation of telomerase RNA localization to Cajal body
0.4
0.6
0.8
1.0
RichFactor
pvalue
0.0005
0.0010
0.0015
0.0020
Count
6
9
12
15
