# Supplementary material for: Multi-Omics Integration Reveals Molecular Signatures Associated with Pork Quality Differences Between Liaodan Black and Xinjin Pigs
Source: Foods. 2026 Oct 15;15(20):3656. doi: 10.3390/foods15203656 (PMC13703621; doi:10.3390/foods15203656)
Supplement: Supplementary file 1 [file foods-15-03656-s001.zip › foods-4607398-supplementary/Supplementary_Materials_Organized/Figures/Figure_S2C_Proteomic_GO_Cellular_Component.pdf]

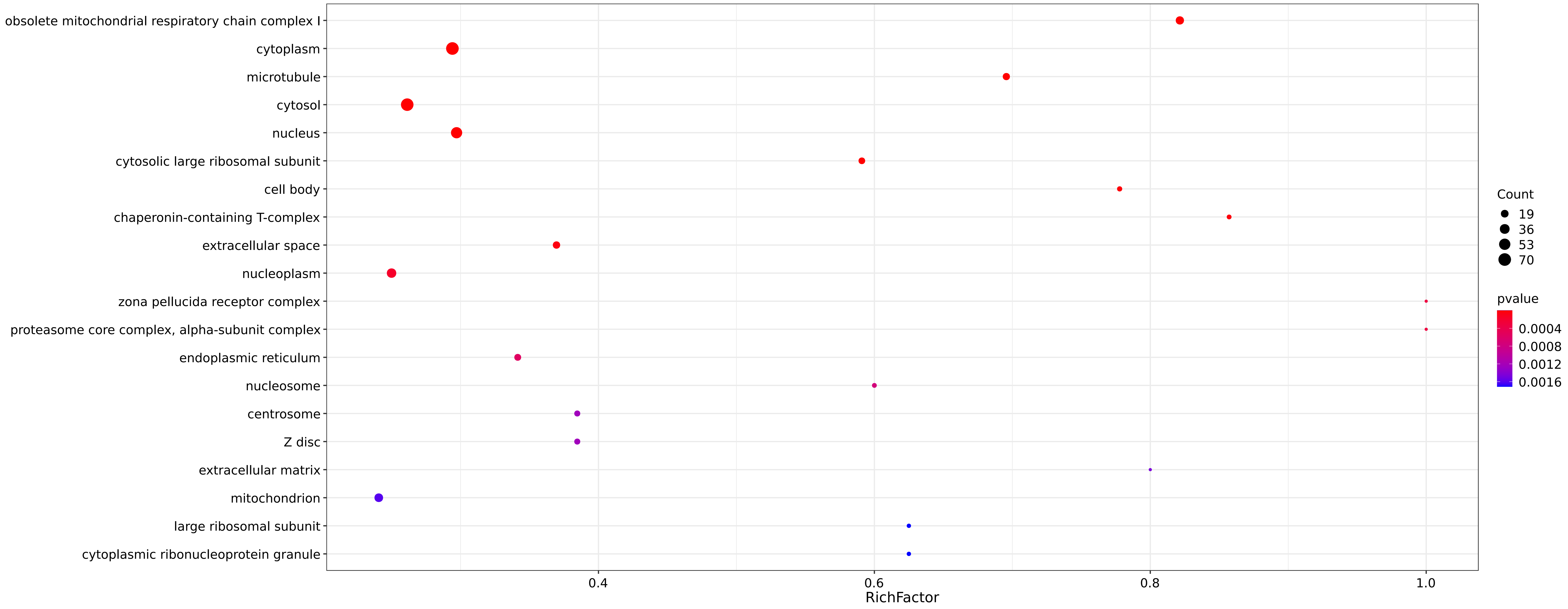

obsolete mitochondrial respiratory chain complex I
cytoplasm
microtubule
cytosol
nucleus
cytosolic large ribosomal subunit
cell body
chaperonin-containing T-complex
extracellular space
nucleoplasm
zona pellucida receptor complex
proteasome core complex, alpha-subunit complex
endoplasmic reticulum
nucleosome
centrosome
Z disc
extracellular matrix
mitochondrion
large ribosomal subunit
cytoplasmic ribonucleoprotein granule
0.4
0.6
0.8
1.0
RichFactor
Count
19
36
53
70
pvalue
0.0004
0.0008
0.0012
0.0016
