# Supplementary material for: Multi-Omics Integration Reveals Molecular Signatures Associated with Pork Quality Differences Between Liaodan Black and Xinjin Pigs
Source: Foods. 2026 Oct 15;15(20):3656. doi: 10.3390/foods15203656 (PMC13703621; doi:10.3390/foods15203656)
Supplement: Supplementary file 1 [file foods-15-03656-s001.zip › foods-4607398-supplementary/Supplementary_Materials_Organized/Figures/Figure_S2D_Proteomic_GO_Molecular_Function.pdf]

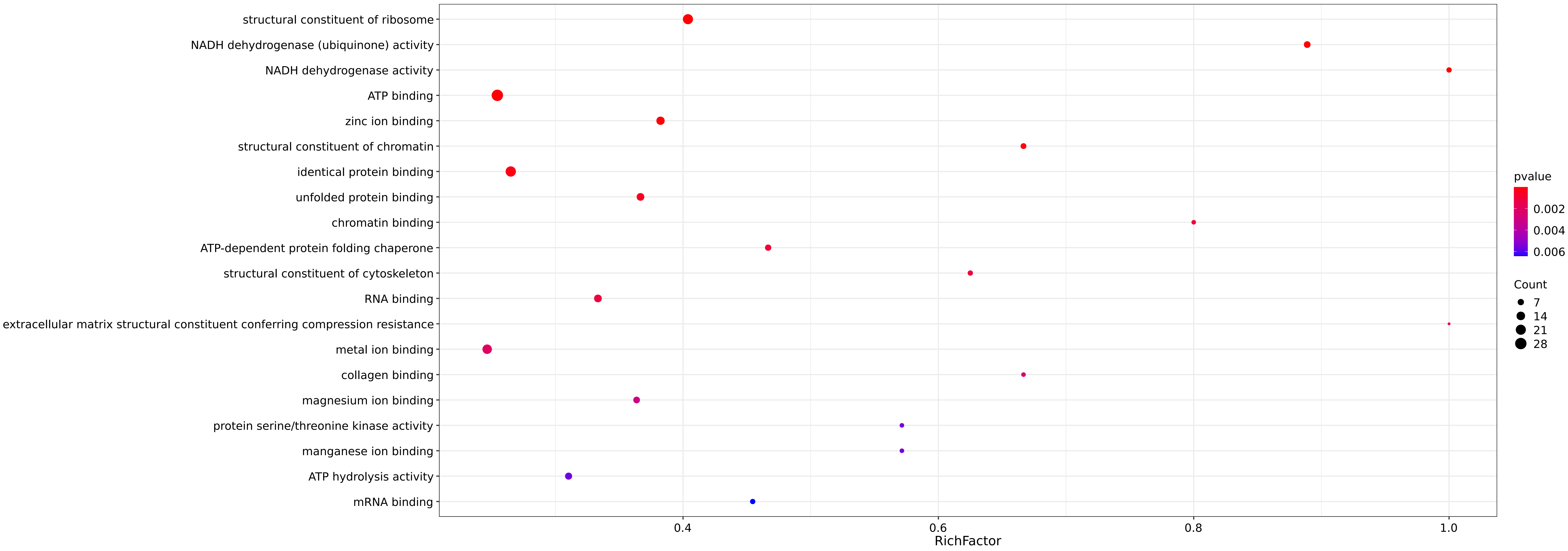
structural constituent of ribosome
NADH dehydrogenase (ubiquinone) activity
NADH dehydrogenase activity
ATP binding
zinc ion binding
structural constituent of chromatin
identical protein binding
unfolded protein binding
chromatin binding
ATP-dependent protein folding chaperone
structural constituent of cytoskeleton
RNA binding
extracellular matrix structural constituent conferring compression resistance
metal ion binding
collagen binding
magnesium ion binding
protein serine/threonine kinase activity
manganese ion binding
ATP hydrolysis activity
mRNA binding
0.4
0.6
0.8
1.0
RichFactor
pvalue
0.002
0.004
0.006
Count
7
14
21
28
