# Supplementary material for: Multi-Omics Integration Reveals Molecular Signatures Associated with Pork Quality Differences Between Liaodan Black and Xinjin Pigs
Source: Foods. 2026 Oct 15;15(20):3656. doi: 10.3390/foods15203656 (PMC13703621; doi:10.3390/foods15203656)
Supplement: Supplementary file 1 [file foods-15-03656-s001.zip › foods-4607398-supplementary/Supplementary_Materials_Organized/Figures/Figure_S3A_Metabolomic_OPLSDA_Positive.pdf]

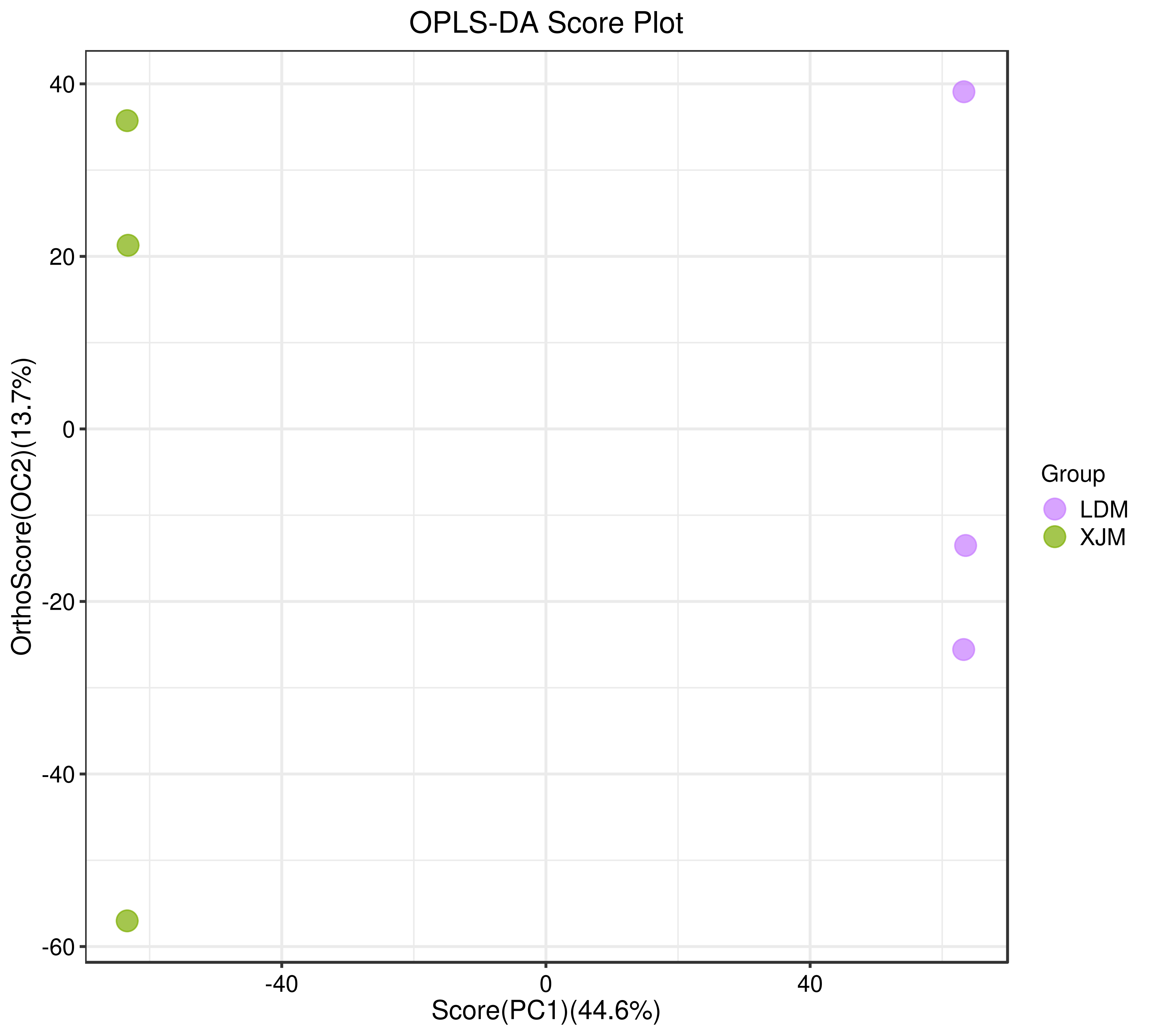
OPLS-DA Score Plot
40
20
0
-20
-40
-60
-40
0
40
Score(PC1)(44.6%)
OrthoScore(OC2)(13.7%)
Group
LDM
XJM
