# Supplementary material for: Multi-Omics Integration Reveals Molecular Signatures Associated with Pork Quality Differences Between Liaodan Black and Xinjin Pigs
Source: Foods. 2026 Oct 15;15(20):3656. doi: 10.3390/foods15203656 (PMC13703621; doi:10.3390/foods15203656)
Supplement: Supplementary file 1 [file foods-15-03656-s001.zip › foods-4607398-supplementary/Supplementary_Materials_Organized/Figures/Figure_S3C_Metabolomic_OPLSDA_Negative.pdf]

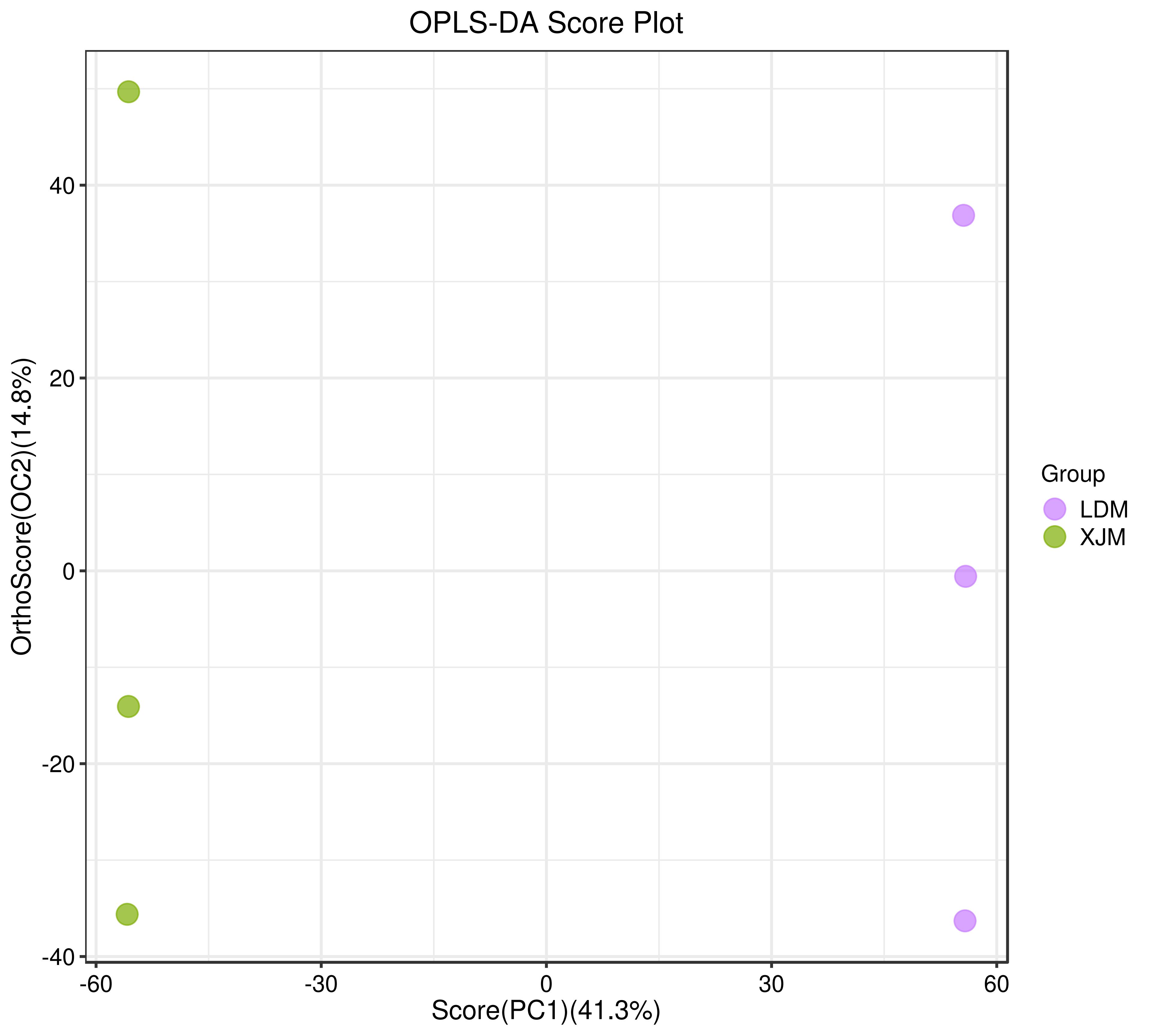
OPLS-DA Score Plot
OrthoScore(OC2)(14.8%)
Score(PC1)(41.3%)
40
20
0
-20
-40
-60
-30
0
30
60
Group
LDM
XJM
