# Supplementary material for: Multi-Omics Integration Reveals Molecular Signatures Associated with Pork Quality Differences Between Liaodan Black and Xinjin Pigs
Source: Foods. 2026 Oct 15;15(20):3656. doi: 10.3390/foods15203656 (PMC13703621; doi:10.3390/foods15203656)
Supplement: Supplementary file 1 [file foods-15-03656-s001.zip › foods-4607398-supplementary/Supplementary_Materials_Organized/Figures/Figure_S3D_Metabolomic_OPLSDA_Permutation_Negative.pdf]

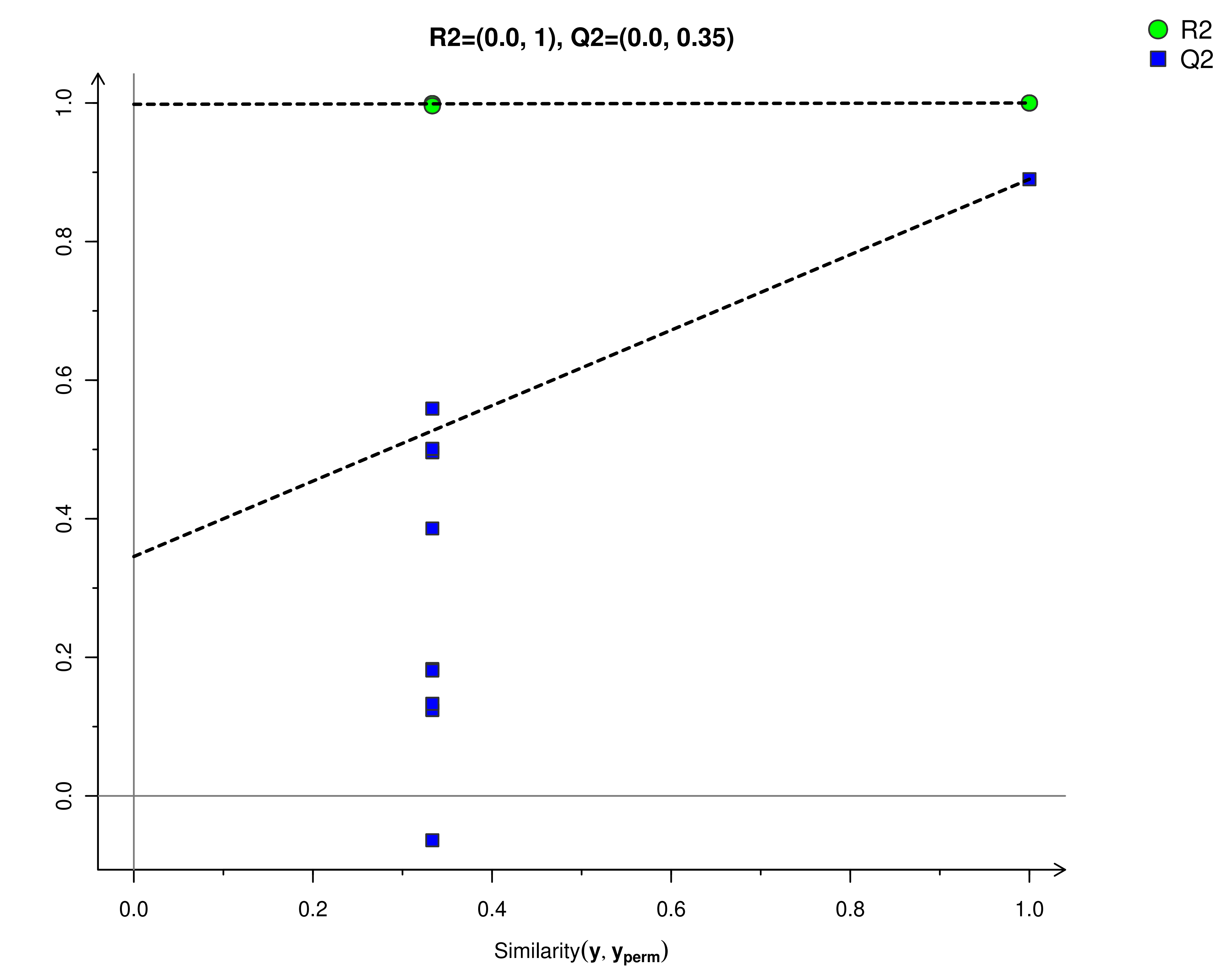

R2=(0.0, 1), Q2=(0.0, 0.35)
R2
Q2
0.0
0.2
0.4
0.6
0.8
1.0
Similarity(y, y_perm)
