# Supplementary material for: Multi-Omics Integration Reveals Molecular Signatures Associated with Pork Quality Differences Between Liaodan Black and Xinjin Pigs
Source: Foods. 2026 Oct 15;15(20):3656. doi: 10.3390/foods15203656 (PMC13703621; doi:10.3390/foods15203656)
Supplement: Supplementary file 1 [file foods-15-03656-s001.zip › foods-4607398-supplementary/Supplementary_Materials_Organized/Figures/Figure_S3E_Metabolomic_Differential_Metabolite_Clustering.pdf]

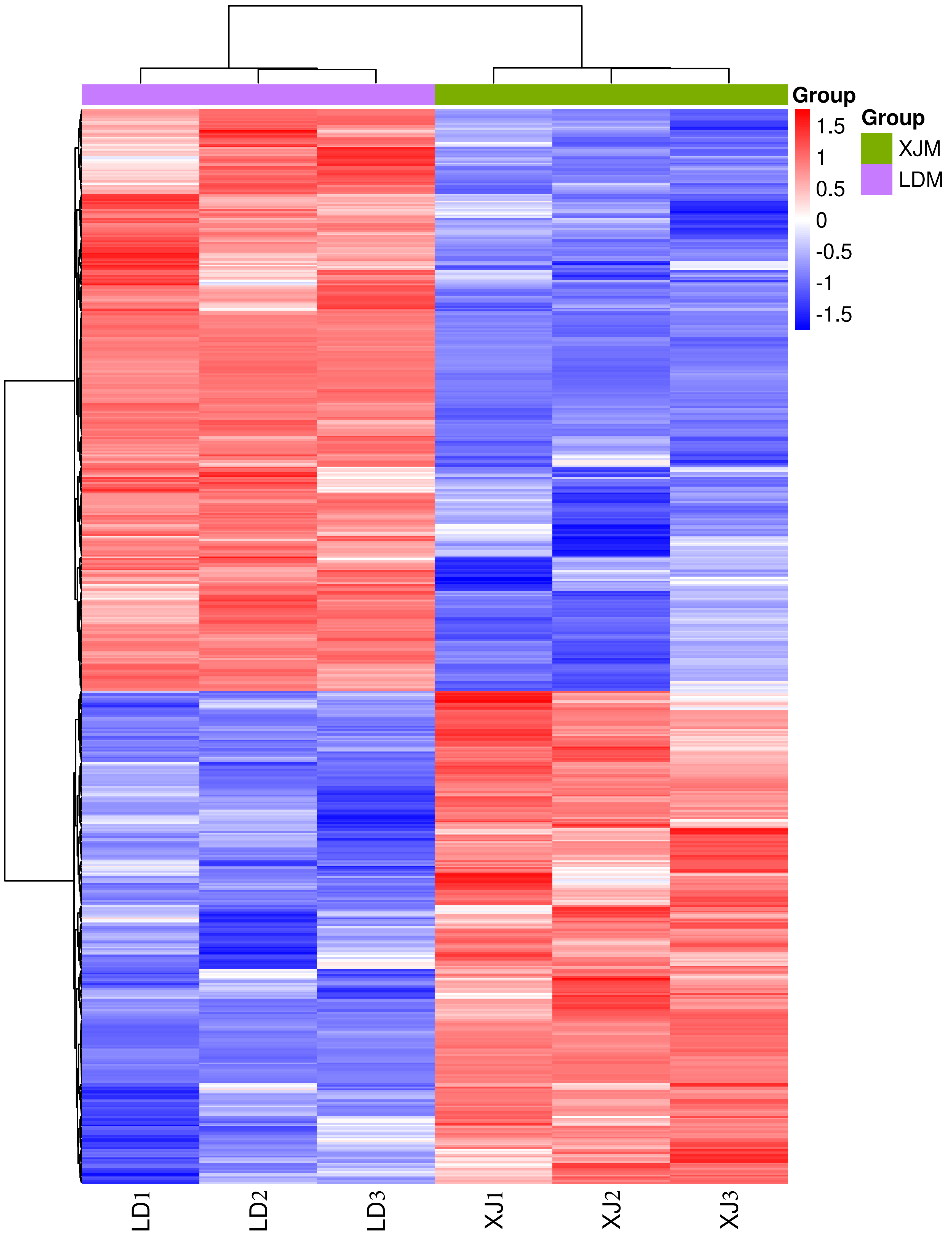
Group
Group
XJM
LDM
1.5
1
0.5
0
-0.5
-1
-1.5
LD1
LD2
LD3
XJ1
XJ2
XJ3
