# Supplementary material for: Multi-Omics Integration Reveals Molecular Signatures Associated with Pork Quality Differences Between Liaodan Black and Xinjin Pigs
Source: Foods. 2026 Oct 15;15(20):3656. doi: 10.3390/foods15203656 (PMC13703621; doi:10.3390/foods15203656)
Supplement: Supplementary file 1 [file foods-15-03656-s001.zip › foods-4607398-supplementary/Supplementary_Materials_Organized/Figures/Figure_S4_RNAseq_DESeq2_Dispersion.pdf]

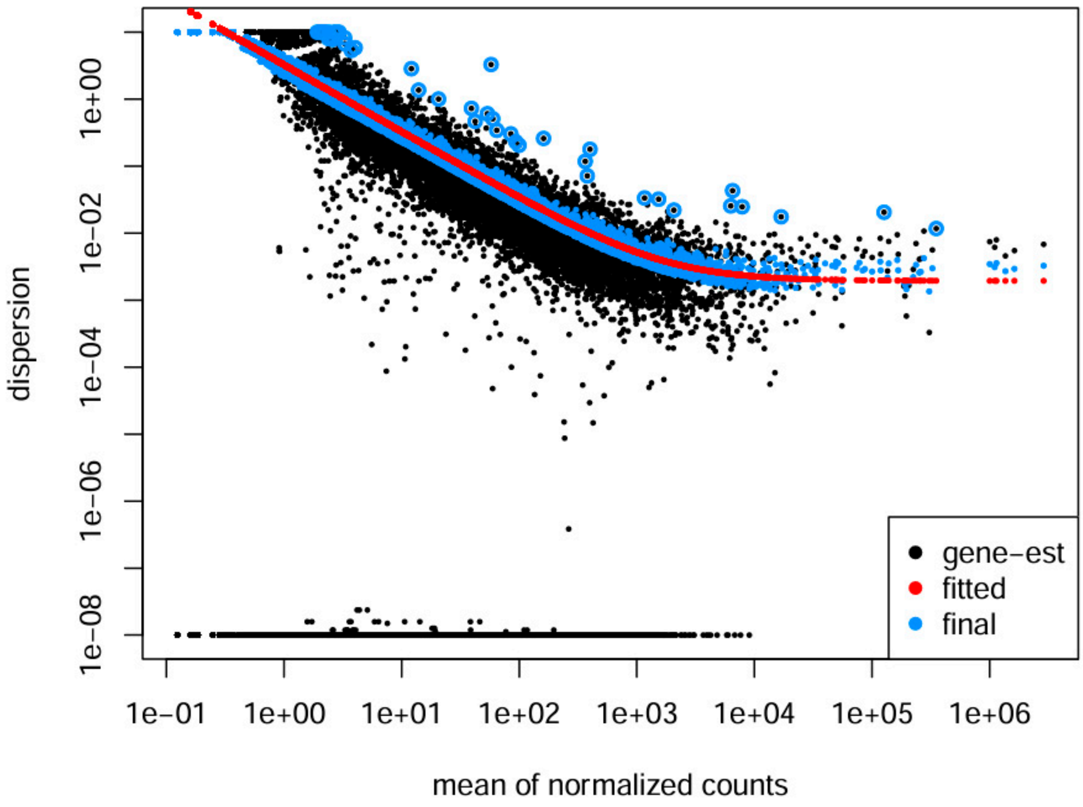

Supplementary Figure S4. DESeq2 dispersion estimates for genes included in the RNA-seq differential-expression analysis. Gene-wise dispersion estimates (black), the fitted mean–dispersion trend (red), and the final dispersion estimates after shrinkage (blue) are shown. The diagnostic was generated from the same raw gene-level count matrix used for differential-expression analysis.
