# Supplementary material for: Multi-Omics Integration Reveals Molecular Signatures Associated with Pork Quality Differences Between Liaodan Black and Xinjin Pigs
Source: Foods. 2026 Oct 15;15(20):3656. doi: 10.3390/foods15203656 (PMC13703621; doi:10.3390/foods15203656)
Supplement: Supplementary file 1 [file foods-15-03656-s001.zip › foods-4607398-supplementary/Supplementary_Materials_Organized/Supplementary_Figure_Legends.pdf]

Supplementary Figure Legends

Figure S1. KEGG pathway enrichment analysis of differentially expressed genes in the LTL muscle of Liaodan Black (LDM) and Xinjin (XJM) pigs (n = 3 per breed). The plot shows 20 pathways, including disease-related pathways not displayed in Figure 2E. Dot size represents the number of mapped differentially expressed genes, and color indicates $-\log_{10}$(q-value).

Figure S2. Additional proteomic analyses of the LTL muscle in Liaodan Black (LDM) and Xinjin (XJM) pigs (n = 3 per breed). (A) Hierarchical clustering heatmap of differentially abundant proteins; (B) GO biological process enrichment analysis; (C) GO cellular component enrichment analysis; (D) GO molecular function enrichment analysis. In panels B–D, dot size represents the number of differentially abundant proteins mapped to each GO term, and color indicates the P-value.

Figure S3. Additional metabolomic analyses of the LTL muscle in Liaodan Black (LDM) and Xinjin (XJM) pigs (n = 3 per breed). (A) Orthogonal partial least squares discriminant analysis (OPLS-DA) in positive-ion mode; (B) permutation test of the OPLS-DA model in positive-ion mode; (C) OPLS-DA in negative-ion mode; (D) permutation test of the OPLS-DA model in negative-ion mode; (E) hierarchical clustering heatmap of differential metabolites.

Figure S4. DESeq2 dispersion estimates for genes included in the RNA-seq differential-expression analysis. Gene-wise dispersion estimates (black), the fitted mean–dispersion trend (red), and the final dispersion estimates after shrinkage (blue) are shown. The diagnostic was generated from the same raw gene-level count matrix used for differential-expression analysis.
